# Effect of covalent functionalisation with isocyanates on the electrochemical properties of exfoliated black phosphorus electrodes

**DOI:** 10.1038/s41598-025-20117-3

**Published:** 2025-10-16

**Authors:** Paweł Jakóbczyk, Anna Dettlaff, Mattia Pierpaoli, Barbara Wójcik, Sławomir Makowiec, Robert Bogdanowicz

**Affiliations:** 1 Faculty of Electronics, Telecommunications and Informatics, Gdańsk University of Technology, 11/12 Narutowicza Str, 80-233 Gdańsk, Poland; 2 Faculty of Chemistry, Gdańsk University of Technology, 11/12 Narutowicza Str, 80-233 Gdańsk, Poland; 3 Department of Nanobiotechnology, Warsaw University of Life Science, Ciszewskiego 8, 02-786 Warsaw, Poland

**Keywords:** Phosphorene, Chemical modification, Electrochemical properties, Liquid exfoliation, Cyclic voltammetry, Materials science, Materials for devices, Sensors and biosensors

## Abstract

This study investigates the covalent functionalisation of few-layer black phosphorus (FLBP) using isocyanates to overcome its inherent susceptibility to oxidation, which limits its application in electrochemical devices. Key synthesis parameters affecting protection efficacy include reactant concentration, ultrasonic irradiation parameters, and solvent selection. While higher reactant concentrations enhance surface coverage, excessive functionalisation impedes interfacial charge transfer. Ultrasonic treatment facilitates optimal reactant-surface interactions, significantly improving electrochemical stability. Comparative solvent analysis indicates acetonitrile outperforms N, N-dimethylformamide in preserving electrochemical performance while enhancing protection against degradation. The research demonstrates that selected isocyanate reactants can effectively passivate the highly nucleophilic three-coordinated phosphorus atoms on FLBP surfaces through the formation of RNHC(O)-P < and RNHC(O)O-P < moieties at reactive surface sites. Electrochemical characterisation reveals that 2xIP(O)(ch)₂_DMF modification provides protection against phosphorus oxidation while maintaining redox functionality. This modification reveals enhanced charge transfer kinetics relative to pristine FLBP or glassy carbon electrodes. The proposed approach widens the electrochemical potential window by eliminating phosphorus oxidation typically observed at 0.65 V while improving charge exchange at the electrode/electrolyte interface. These findings establish a systematic approach for FLBP stabilisation via isocyanate functionalisation, expanding potential applications in energy storage systems, electrocatalysis, and electrochemical sensing technologies.

**Supplementary Information:**

The online version contains supplementary material available at 10.1038/s41598-025-20117-3.

## Introduction

In recent years, the intriguing realm of two-dimensional (2D) materials has captivated researchers around the world due to their unusual properties, which differ significantly from their bulk counterparts^1–3^. Among these materials, two-dimensional black phosphorus (BP), also known as phosphorene, has emerged as a promising elemental analogue of graphene^1,4,5^. However, BP offers a broader range of functional properties than graphene due to its anisotropy, a thickness-dependent band gap ranging from 0.3 eV to 2.0 eV, and remarkable carrier mobilities of approximately 1000 cm^2^ V^-^¹ s^-^¹. Additionally, its optical and thermal transport properties can be tailored by crystal orientation^4,6,7^. With relatively weak interlayer van der Waals forces and strong in-plane bonding, BP can be easily exfoliated, similar to graphene and other transition metal dichalcogenides (TMDs)^8,9^. It was observed that not only the monolayer structure of black phosphorus exhibits intriguing properties, but multilayer structures (2–10 layers) also demonstrate notable characteristics^10–12^. Wieloszynska in her work^13^ shows that even a 38.4 nm layer of differently oriented flakes has the resultant properties of single layer phosphorene and not those of bulk black phosphorus. The wide range of applications for BP includes energy storage devices^14–16^, field effect transistors^17,18^, thermoelectrics^19,20^, broadband photodetectors^21,22^, various electrochemical sensors^23,24^, including biosensors^25–27^.

However, the long-term stability of phosphorene is severely compromised by the presence of densely packed lone-pair electrons on its surface^28–31^, which serve as favourable chemisorption sites for oxygen-containing species. As a result, a hydrogen bond forms between the surface-chemisorbed oxygen and hydrogen from water, ultimately leading to the breaking of the P-P bond^32^. This makes BP highly susceptible to deterioration, especially in high anodic potential environments, for example, during the oxygen evolution reaction (OER) processes^33^.

To address this limitation, various passivation strategies have been investigated, including both non-covalent approaches (e.g., surfactant coatings, encapsulation) and covalent modifications involving radical chemistry^34^, diazonium salts^35^, iodonium salts, halides^36^, organometallic reagents^37,38^, azides^39^, or Lewis acid/base adducts^40^. While covalent functionalization can effectively stabilize the BP surface by altering its electronic environment, many such reactions require harsh conditions or result in structural damage due to lattice cleavage. A detailed comparative overview of the main covalent passivation strategies for black phosphorus is provided in Table S1, SI 1, highlighting key differences in reactive groups, bond types, stability, and functional tunability. Notably, the isocyanate-based approach introduced in this work combines high chemical, electrochemical and thermal stability with broad functional flexibility under mild, catalyst-free conditions—without disrupting the P–P lattice. The formation of covalent bonds redistributes electrons on the catalyst surface, modifying the local electronic structure of the active sites via the ligand effect^35^. In particular, strong coordination bonds between metal species and coordinating atoms with single-pair electrons such as P, S, N and O can significantly improve catalytic stability for long-term applications^41^. Thus, these passive covalent functionalisation strategies hold great promise for improving the electrocatalytic performance of BP^33^. In this study, we explore the modification of black phosphorus multilayers using isocyanates, a highly electrophilic species known to react with various nucleophilic reagents, including phosphorus^42–44^. Isocyanates represent a broad and versatile class of compounds, which allows for surface modification of FLBP in various directions by significantly altering its physicochemical properties. Drawing from literature reports highlighting the beneficial role of isocyanates in forming uniform and stable solid electrolyte interphases (SEI) in lithium-ion batteries, similar functionalization of phosphorene could be explored to enhance interfacial stability in electrochemical systems. Moreover, by selecting appropriately substituted isocyanates, it is possible to tailor the surface polarity of FLBP-based electrodes, which may improve sensor performance by promoting selective interactions with specific analytes in solution—thus enhancing the sensitivity of the device toward those target compounds. These are only a few selected examples of potential applications, and the functionalization potential of isocyanates in 2D material chemistry remains broad and largely unexplored.

Given that the surface of phosphorene consists of highly nucleophilic three-coordinated P atoms, we selected a series of isocyanates for this modification without the need for additional catalysts. The modification of few-layer black phosphorus (FLBP) with isocyanates presents a novel approach to enhancing its stability and electrochemical properties, thereby expanding its potential utility across various applications, especially in electrochemistry.

## Experimental section

### Chemicals

Black phosphorus (BP, > 99.99%) was obtained from Smart Elements. N, N-dimethylformamide (DMF), anhydrous, purity: 99.5% and acetonitrile (ACN), anhydrous, purity: 99.8% were collected from Sigma Aldrich. Commercially available isocyanates: ethyl isocyanate (purity: 98%), 2-chloroethyl isocyanate (purity: 96%), *p*-tolyl isocyanate (purity: 99%), chloroacetyl isocyanate (purity: 97%), trichloroacetyl isocyanate (purity: ≥ 95%), and isocyanatophosphonic dichloride (purity: 95%) – were purchased from Merck. The potassium chloride KCl (ACS reagent, purity ≥ 99%, Aldrich), potassium hexacyanoferrate (III) K_3_[Fe(CN)_6_], potassium hexacyanoferrate (II) trihydrate K_4_[Fe(CN)_6_] × 3H_2_O (pure p.a., Chempur), and hydrochloric acid HCl (35–38%, Chempur) aqueous solutions were prepared using demineralised water. The argon was collected from Air Liquide and was of the highest purity class. Two isocyanate derivatives, 4-(chloromethyl)benzoyl azide and 4-(chloromethyl)phenyl isocyanate, were synthesised prior to use.

To synthesise 4-(chloromethyl)benzoyl azide, 4-(chloromethyl)benzoic acid (3.41 g, 20 mmol, Sigma-Aldrich), diphenyl phosphoryl azide (6.05 g, 22 mmol, Sigma-Aldrich), and triethylamine (3.06 mL, 22 mmol, Sigma-Aldrich) were dissolved in anhydrous dioxane (20 mL, Sigma-Aldrich). The reaction mixture was stirred at room temperature for 1 hour, after which the solvent was removed under reduced pressure (T < 30 °C). The resulting residue was dissolved in diethyl ether (50 mL), washed successively with demineralised water (3 × 15 mL) and brine (2 × 10 mL), dried over anhydrous MgSO₄, filtered, and concentrated under reduced pressure to yield 4-(chloromethyl)benzoyl azide as a white solid (3.88 g, 99%). ^1^H NMR (CDCl_3_, 500 MHz): δ = 8.03 (d, J = 8.4 Hz, 2 H), 7.49 (d, J = 8.4 Hz, 2 H), 4.62 (s, 2 H); ^13^C NMR (CDCl_3_, 125 MHz): δ = 171.99, 143.74, 130.55, 129.90, 128.75, 45.18; FT-IR (ATR) 2132 cm^−1^ N = N = N, 1685 cm^−1^ C = O. Spectral data are provided in the Supplementary Materials (SI 2).

For the synthesis of 4-(chloromethyl)phenyl isocyanate, the crude 4-(chloromethyl)benzoyl azide (3.88 g, 19.9 mmol) was dissolved in dry dioxane (20 mL) and heated in an oil bath at 100 °C for 2 h. Upon completion of the Curtius rearrangement, indicated by the cessation of nitrogen evolution, the solvent was removed under reduced pressure to afford 4-(chloromethyl)phenyl isocyanate as a yellow oil. ^1^H NMR (CDCl_3_, 500 MHz): δ = 7.36 (d, J = 8.4 Hz, 2 H), 7.10 (d, J = 8.4 Hz, 2 H), 4.56 (s, 2 H); ^13^C NMR (CDCl_3_, 125 MHz): δ = 135.08, 133.53, 130.08, 129.87, 125.02, 45.49; FT-IR (ATR) 2240 cm^−1^ N = C = O. Spectral data are provided in the Supplementary Materials (SI 2).

### Few-layer black phosphorus solution preparation

The FLBP solution was prepared following the same procedure as detailed in our previous publication^45^. FLBP was obtained through solvent-assisted exfoliation of 45 mg of pre-ground black phosphorus. The BP was dispersed in 7.5 mL of deoxygenated DMF by purging with argon. The BP dispersion underwent sonication under an argon stream using a horn probe ultrasonicator (Bandelin Sonopuls HD2200, 20 kHz), maintaining a temperature range of 0 to 3 °C with an ice-cooled bath. The sonication tip operated at 40 W power with a 0.5/0.5 s on/off time for 4 h to disrupt the van der Waals bonds in the BP crystal.

### Functionalisation of FLBP with isocyanates

The selection of specific isocyanates for FLBP surface modification was guided by both their chemical reactivity and structural diversity. We aimed to explore how variations in steric hindrance, electronic properties, and functional group compatibility affect the efficiency and stability of surface bonding. The isocyanates tested included simple alkyl (e.g., ethyl), aryl (*p*-tolyl), haloalkyl (e.g., 2-chloroethyl), acyl-substituted (chloroacetyl, trichloroacetyl), and phosphonic derivatives. In addition, halo-substituted isocyanates bearing electrophilic centres were chosen for their dual reactivity, acting both as alkylating agents and as enhancers of the -NCO group electrophilicity.

**Fig. 1:**
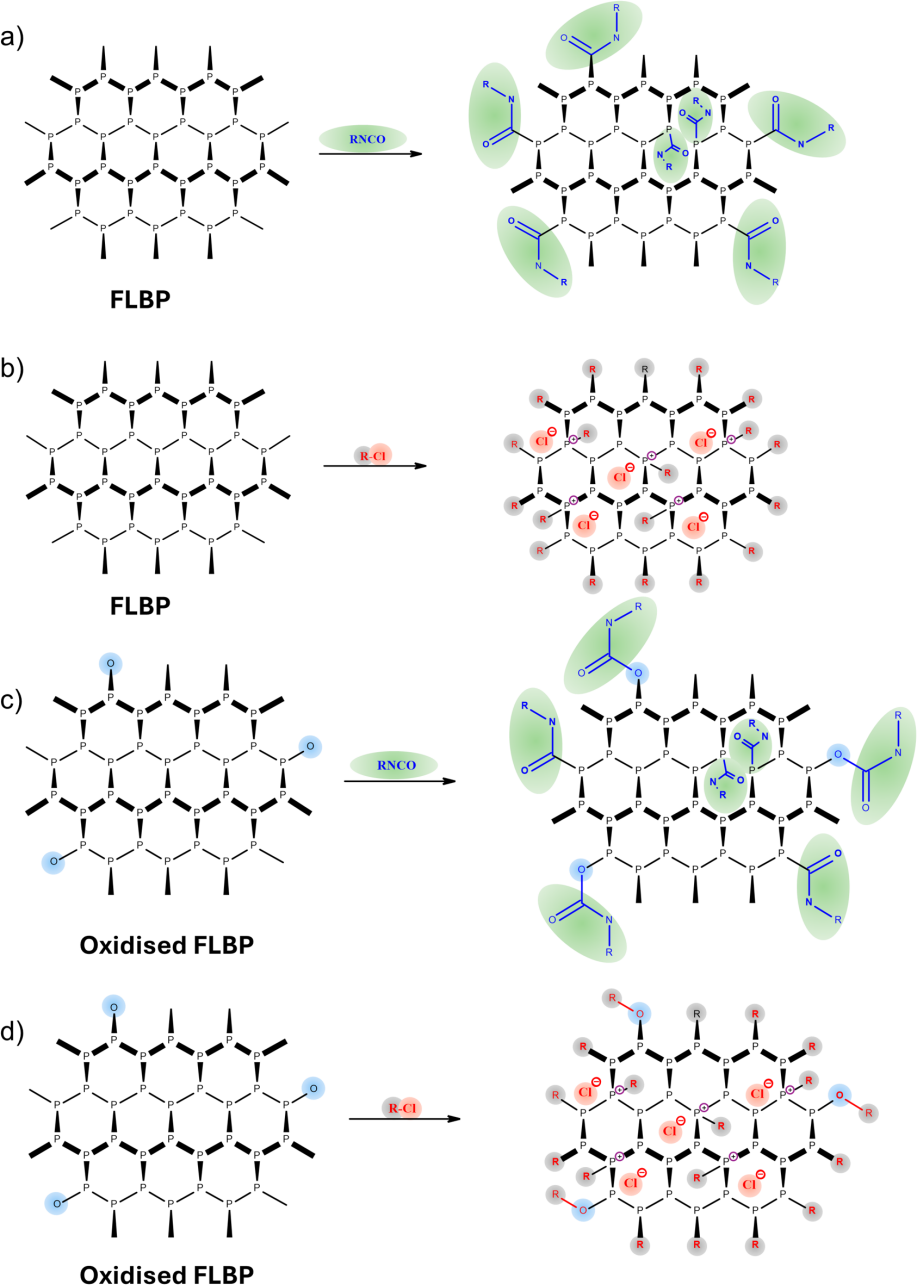
Schematic representation of FLBP suspension modification using (a) RNCO, (b) R-Cl, and oxidised FLBP modification using (c) RNCO, (d) R–Cl.

The surface protection concept relied on the formation of covalent bonds between the FLBP surface or partially oxidised FLBP and isocyanate reagents, specifically through the creation of RNHC(O)–P < or RNHC(O)O–P < motifs (Fig. 1a, c). These motifs are expected to form at reactive sites, namely dislocation defects and edge sites of FLBP, which contain phosphorus atoms capable of releasing a bond from the lattice structure. These sites are also the most vulnerable to environmental degradation via oxidation or hydrolysis. The incorporation of isocyanates with hydrophobic and sterically bulky substituents was designed not only to stabilise these active sites through covalent bonding but also to shield nearby regions via non-covalent, steric “molecular umbrellas”. This protective effect is especially crucial in aqueous or oxidative environments, where rapid degradation of unmodified FLBP surfaces typically occurs.

Besides isocyanates, we also explored the reactivity of acid chlorides and alkyl halides with FLBP. Acid chlorides were chosen due to their expected similar reaction kinetics and mechanism (nucleophilic attack by phosphorus). In contrast, alkyl halides were included as a less selective modification route, potentially forming phosphonium-type salts (≡ P⁺–R) on the surface (Fig. 1b, d). Hybrid compounds, such as halo-substituted isocyanates, were particularly interesting due to their ability to engage in both electrophilic addition and halogen-driven reactivity. For instance, in perhalogenated derivatives, the halogens are unlikely to act independently as alkylating agents, but they significantly enhance the NCO group’s reactivity.

Detailed descriptions of the FLBP modifications using the tested reagents and various synthesis parameters are provided in the Supplementary Information (Section SI 3). Summary information on the types of modifications is given in Table 1. All chemical formulas of the reagents used for FLBP modification are provided in the Supplementary Materials (SI 4). An explanation and understanding of the abbreviations used in the manuscript are provided in the Supplementary Materials (SI 5).

**Table 1:** Parameters used for FLBP suspension modification.

PM	Symbol of the electrode	Modification of FLBP	Synthesis conditions*	US	Ratio FLBP: ISO RNCO
1	EI_DMF	C_2_H_5_NCO- Ethyl isocyanate	Method A	N	1:1
2	2chEI_DMF	ClC_2_H_4_NCO- 2-Chloroethyl isocyanate	Method A	N	1:1
3	p-MphI_DMF	p-CH_3_C_6_H_4_NCO- p-tolyl isocyanate	Method A	N	1:1
4	p-chMphI_DMF	p-ClCH_2_C_6_H_4_NCO-p-(Chloromethyl)phenyl isocyanate	Method A	N	1:1
6	2xEI_DMF_US	C_2_H_5_NCO- ethyl isocyanate	Method B	Y	1:2
7	2 × 2chEI_DMF_US	ClC_2_H_4_NCO- 2-Chlroethyl isocyanate	Method B	Y	1:2
8	2xp-MphI_DMF_US	p-CH_3_C_6_H_4_NCO- p-tolyl isocyanate	Method B	Y	1:2
9	2xp-(chM)phI_DMF_US	p-ClCH_2_C_6_H_4_NCO- p-(Chloromethyl)phenyl isocyanate	Method B	Y	1:2
10	2xchAI_DMF	ClCH_2_C(O)NCO-Chloroacetyl isocyanate	Method C	N	1:2
11	2x(ch)_3_AI_DMF	Cl_3_CC(O)NCO-Trichloroacetyl isocyanate	Method C	N	1:2
12	2xIP(O)(ch)_2__DMF	Cl_2_P(O)NCO-Isocyanatophosphonic dichloride	Method C	N	1:2
13	2xchAch_DMF	ClCH_2_C(O)Cl- Chloroacetyl chloride	Method C	N	1:2
14	EI_mDMF	C_2_H_5_NCO- Ethyl isocyanate	Method A’	N	1:1
15	EI_mDMF_US	C_2_H_5_NCO- Ethyl isocyanate	Method D	Y	1:1
16	EI_mDMF	C_2_H_5_NCO- Ethyl isocyanate	Method A’	N	1:1
17	EI_mDMF_US	C_2_H_5_NCO- Ethyl isocyanate	Method D	Y	1:1
18	2chEI_mDMF	ClC_2_H_4_NCO- 2-Chloroethyl isocyanate	Method A’	N	1:1
19	2chEI_mDMF_US	ClC_2_H_4_NCO- 2-Chloroethyl isocyanate	Method D	Y	1:1
20	2chEI_mDMF	ClC_2_H_4_NCO- 2-Chloroethyl isocyanate	Method A’	N	1:1
21	2chEI_mDMF_US	ClC_2_H_4_NCO- 2-Chloroethyl isocyanate	Method D	Y	1:1
22	1chB_DMF	C_4_H_9_Cl- 1-chlorobutane	Method A’	N	1:1
23	1chB_mDMF_US	C_4_H_9_Cl- 1-chlorobutane	Method D	Y	1:1
24	1chB_mDMF	C_4_H_9_Cl- 1-chlorobutane	Method A’	N	1:1
25	1chB_mDMF_US	C_4_H_9_Cl- 1-chlorobutane	Method D	Y	1:1
26	chAI_DMF	ClCH_2_C(O)NCO- Chloroacetyl isocyanate	Method A	N	1:1
27	(ch)_3_AI_DMF	Cl_3_CC(O)NCO- Trichloroacetyl isocyanate	Method A	N	1:1
28	2chEI_DMF	ClC_2_H_4_NCO- 2-Chloroethyl isocyanate	Method A	N	1:1
29	2xchAI_DMF	ClCH_2_C(O)NCO- Chloroacetyl isocyanate	Method C	N	1:2
30	2x(ch)_3_AI_DMF	Cl_3_CC(O)NCO- Trichloroacetyl isocyanate	Method C	N	1:2
31	2 × 2chEI_DMF	ClC_2_H_4_NCO- 2-Chloroethyl isocyanate	Method C	N	1:2
32	2xchAI_ACN	ClCH_2_C(O)NCO- Chloroacetyl isocyanate	Method E	N	1:2
33	2x(ch)_3_AI_ACN	Cl_3_CC(O)NCO- Trichloroacetyl isocyanate	Method E	N	1:2
34	2 × 2chEI_ACN	ClC_2_H_4_NCO- 2-Chloroethyl isocyanate	Method E	N	1:2
35	0.5 × 2chEI_DMF	ClC_2_H_4_NCO- 2-Chloroethyl isocyanate	Method F	N	1:0.5
36	2chEI_DMF	ClC_2_H_4_NCO- 2-Chloroethyl isocyanate	Method A	N	1:1
37	2 × 2chEI_DMF	ClC_2_H_4_NCO- 2-Chlroethyl isocyanate	Method C	N	1:2
38	0.5xchAI_DMF	ClCH_2_C(O)NCO- Chloroacetyl isocyanate	Method F	N	1:0.5

US – ultrasonic treatment

*A detailed description of the Methods used for the modifications can be found in the Supplementary Materials (SI 3).

### FLBP and modified FLBP electrode preparation

For the measurements, the electrode film was formed on glassy carbon electrodes (GCE) using previously modified FLBP suspensions. The film preparation involved two main steps. First, 20 µL of the modified (or unmodified) FLBP slurry was drop-cast onto the glassy carbon disc electrode in an argon-filled glovebox. The FLBP loading on the electrode depends on the modification method (SI 3: Methods of modification) used and ranges from approximately 0.9 to 1.5 mg·cm^-^². The electrode was then left to dry slowly in the glovebox for 24 h. After this initial drying step, it was transferred to a vacuum oven (also within the glovebox) and further dried under vacuum at 40 °C for an additional 12 h.

### Characterisation methods

#### TEM

The topography of FLBP and modified FLBP was visualized using a JEM-1220 transmission electron microscope (TEM; JEOL, Tokyo, Japan) operating at 80 kV and equipped with a Morada 11-megapixel camera (Olympus Soft Imaging Solutions, Münster, Germany).

#### Raman spectroscopy

Raman measurements were performed using a confocal XploRA PLUS microspectrometer (HORIBA Scientific) equipped with 532 nm and 785 nm excitation lasers. The system featured a fully automated spectrometer with four diffraction gratings (600–2400 gr/mm) and a thermoelectrically air-cooled CCD detector (1024 × 256 px). Spectral resolution was ≤ 1.4 cm⁻¹ for 532 nm and ≤ 1.2 cm⁻¹ for 785 nm.

#### FTIR

Fourier transform infrared (FTIR) spectra of FLBP and its modifications were obtained using a Bruker Invenio R spectrometer equipped with a Ge-ATR unit (Bruker Optik GmbH, Ettlingen, Germany). The Ge-ATR FTIR spectra were recorded in the 4000–550 cm^−1^ range.

#### NMR

1 H and 13 C NMR spectra were recorded with Varian Gemini 500 MHz and NMR chemical shifts were reported in δ (ppm) using residual solvent peaks as standards, with the coupling constant J measured in Hz.

#### Electrochemical measurements

The electrochemical behaviour of FLBP and modified FLBP was carried out using a potentiostat-galvanostat (VMP-300, Bio-Logic, France) with EC-Lab (V 11.50) software. Measurements were performed in a three-electrode configuration (Pt wire – counter electrode, Ag/AgCl wire – pseudo-reference electrode) in an inert atmosphere. FLBP and modified FLBP drop-cast onto a commercially available glassy carbon (GC) electrode (ϕ = 3 mm, Mineral, Poland) were used as the working electrodes. Prior to each measurement, the GC disc electrodes were polished with an Al_2_O_3_ slurry using a MicroCloth polishing pad (Buehler, USA), followed by ultrasonic cleaning in deionized water to remove any residual polishing material. Electrochemical measurements were performed in triplicate (*n* = 3), where *n* = 3 refers to measurements carried out on three independently prepared electrodes (separate deposited inks). Electrochemical measurements were taken outside the glovebox, but the electrolyte was deoxygenated with argon for a minimum time of 7 min before the electrodes were immersed in it. The kinetic performance of the coatings was tested using cyclic voltammetry (CV) and electrochemical impedance spectroscopy (EIS) in the one-electron transfer inner sphere redox mediator 5 mM [Fe(CN)_6_]^3-/4-^ in 1 M KCl, The 5 mM [Fe(CN)_6_]^3-/4-^ solution was prepared by mixing two salts K_3_[Fe(CN)_6_] (2.5 mM) and K_4_[Fe(CN)_6_] (2.5 mM). Electrochemical impedance spectroscopy was additionally recorded in supporting electrolyte alone (1 M KCl). CV tests were carried out in the potential range between − 0.25 V and + 0.7 V (vs. Ag/AgCl immersed in 1 M KCl solution) as a function of scan rates: 5 mV s^−1^, 10 mV s^−1^, 25 mV s^−1^, 50 mV s^−1^, 100 mV s^−1^, 150 mV s^−1^, 200 mV s^−1^, 300 mV s^−1^. EIS was conducted by measuring the electrical response of the system at its resting potential. Measurements were made over a wide frequency range, spanning from 0.1 Hz to 100 kHz, with an amplitude of 10 mV. The electrode was exposed to a fixed voltage value of open circuit potential for 20 min before the impedance spectra were measured. Spectra in the mediator were fitted with a Randles-type EEQC comprising R_s_ in series with (CPE∥R_ct_) and a finite-length Warburg element, as detailed in Fig. S2 (SI 8).

## Results and discussion

### Physicochemical characterisation of modified FLBP

**Fig. 2:**
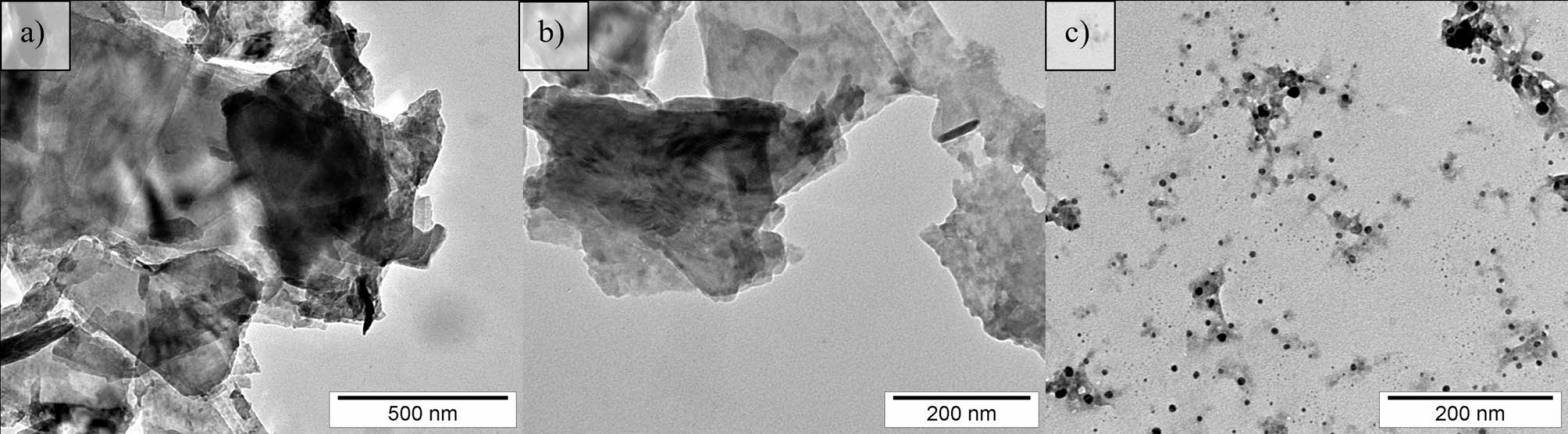
TEM images of (a) and (b) FLBP and FLBP modified with 2xIP(O)(ch)_2__DMF.

The TEM images presented in Fig. 2 illustrate the topography of FLBP before and after chemical modification. Figure 2a and b show pristine FLBP, which exhibits a characteristic thin, sheet-like topography. The flakes have lateral dimensions on the order of several hundred nanometers, with sharp, well-defined edges and uniform electron transparency. This structure indicates high crystallinity and minimal surface contamination, consistent with typical properties of unmodified FLBP reported in the literature^39^. Figure 2c presents a sample of modified FLBP (2xIP(O)(ch)_2__DMF), which was selected due to its superior electrochemical performance among the studied modifications. In this image, distinct morphological changes can be observed: the flakes are smaller in lateral size, their edges appear more diffuse, and the surface texture is more granular and irregular. These features suggest successful covalent surface modification, likely involving the formation of a functional layer. Additionally, the reduced flake size may be partially attributed to intense stirring during the modification process, which could contribute to fragmentation.

Based on the comparison of the Raman spectra of pristine FLBP and covalently modified FLBP (2×IP(O)(CH)_2__DMF), the three characteristic Raman bands assigned to the *A*^*1*^_*g*_, *B*_*2g*_, and *A*^*2*^_*g*_ vibrational modes exhibit noticeable blue shifts. Specifically, the *A*^*1*^_*g*_ mode shifts from 355 to 360 cm⁻¹, the *B*_*2g*_ mode from 430 to 436 cm⁻¹, and the *A*^*2*^_*g*_ mode from 456 to 463 cm⁻¹. These shifts, observed within the 300–600 cm⁻¹ spectral range, indicate a stiffening of vibrational modes, which is consistent with a strengthening of interatomic bonds in the phosphorus lattice^46–48^. This behaviour suggests that the covalent modification alters the local bonding environment, possibly through structural rearrangements or electronic effects, while the layered architecture of FLBP remains preserved^49^ (see Supporting Information, Figure S1, SI 6).

**Fig. 3:**
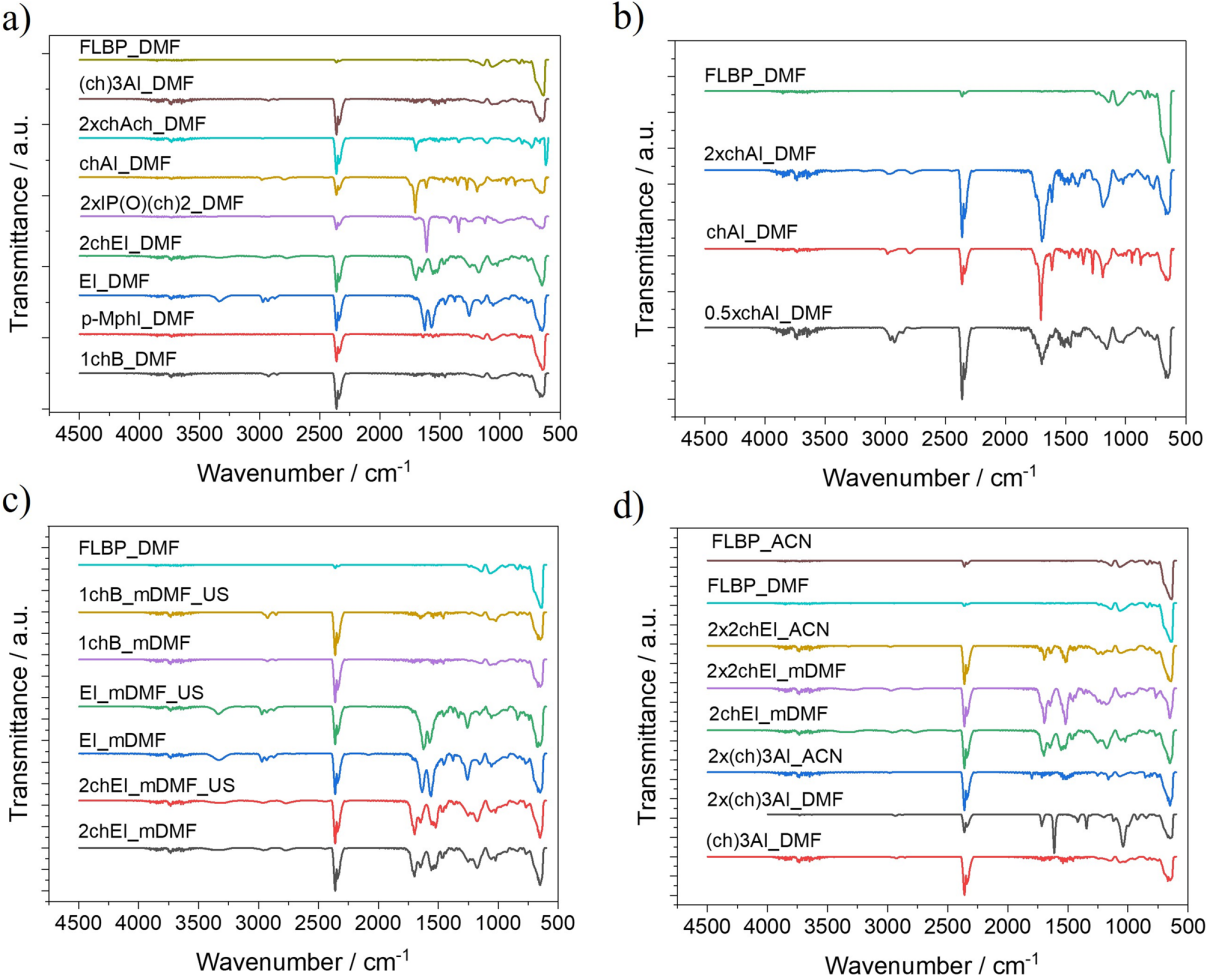
FTIR spectra of modified FLBP: (a) with different chemical compounds, in various concentrations, (b) with and (c) without ultrasonication during synthesis, (d) in different solvents.

The FTIR spectra (Fig. 3a-d) show that the peaks of FLBP and its modifications appear at the same positions around 550–670 cm^−1^. These peaks do not originate from the phosphorus itself but from PO_4_^3−^ ions, for which there are four types of vibrations: O-P-O deformational bending vibrations, O-P-O scissors deformational vibrations, P-O stretching asymmetric vibrations and P-O stretching symmetrical vibrations. Only the bending vibration of the O-P-O bond and the stretching asymmetric vibration of the P-O bond are active and occur between 500 and 564 and 1009 and 1080 cm^−1^, respectively^50^. Figure 3a shows the FTIR spectra of FLBP modified with compounds of varying reactivity. For 1-chlorobutane, there are no distinct peaks corresponding to the CH₂ groups in the 2850–2960 cm⁻¹ range, nor are there peaks observed below 1500 cm⁻¹ (specifically in the 1280–1500 cm⁻¹ range)^51^. Similarly, the FTIR spectrum for the modification with p-methylphenyl isocyanate does not exhibit any characteristic peaks for this compound in the 1000–1600 cm⁻¹ range, suggesting that no reaction has occurred with p-methylphenyl isocyanate^52^. The peak at 2300 cm⁻¹ is clearly attributable to CO₂, most likely arising from ambient carbon dioxide in the optical path^53^. In contrast, for the aliphatic isocyanate (ethyl isocyanate), a low-intensity peak around 2900 cm⁻¹ is observed, which corresponds to the stretching vibrations of the aliphatic C-H bond. The reaction of FLBP with ethyl isocyanate is further confirmed by a visible peak at 3400 cm⁻¹, attributed to the N-H bond, and by the stretching vibrations of the C = O bond in the 1500–1600 cm⁻¹ range. A peak at 1300 cm⁻¹, characteristic of C-N stretching vibrations, is also present. This peak is typical for single C-N bonds and may shift slightly depending on the specific molecular environment and substituents on the nitrogen or carbon atoms. Generally, C-N stretching vibrations are observed in the 1020–1200 cm⁻¹ range^54^.

The reaction of FLBP with chloroethyl isocyanate also appears to have occurred, as evidenced by peaks similar to those observed for ethyl isocyanate^55^. The presence of chlorine in chloroethyl isocyanate may influence the spectral characteristics, although specific chlorine-related peaks are not visible in the FTIR spectra. However, chlorine’s presence Likely affects the shifts observed in the carbonyl group peaks. The FTIR spectra of the obtained compound 2xIP(O)(ch)₂_DMF show characteristic absorption bands for the C = O (1615 cm⁻¹) and P = O (1200–1260 cm⁻¹) groups. A notable change is observed in the PO₄³⁻ peak for 2xIP(O)(ch)_2__DMF, where the shape of the peaks has changed and their intensity is lower, suggesting that the compound protects against the formation of PO_4_^3−^. There is also a significant shift in the PO₄³⁻ peak region for chAI_DMF, which further confirms the reduced oxidation of FLBP due to the reaction with chAI_DMF^52^. Additionally, the FTIR spectra of 2chAch_DMF and (ch)_3_AI_DMF are shown in Fig. 3a. Both compounds exhibit similar FTIR spectra, which would be expected to show significant reactivity. However, the spectral changes compared to FLBP_DMF (the starting material) are negligible in the PO₄³⁻ region and within the 1450–1750 cm⁻¹ range, suggesting that no reaction has occurred with these compounds. The concentration of isocyanate only has an effect when the amount of reagent is insufficient. In such cases the ratio of the heights of the peaks coming from the oxidised FLBP and carbonyl groups varies, a higher carbonyl peak being observed at higher isocyanate concentrations (2xchAI_DMF and chAI_DMF) and a lower peak at 0.5xchAI_DMF (Fig. 3b)^52,55^. It can also be concluded that the presence of ultrasound during the reaction in DMF does not affect the resulting product. The FTIR spectra for 1chB_mDMF_US and 1chB_mDMF, as well as for EI_mDMF_US and EI_mDMF, and 2chEI_mDMF_US and 2chEI_mDMF show no differences between the reactions carried out with and without ultrasound (Fig. 3c). Regarding the influence of the solvent on the product of the FLBP reaction with isocyanate, more intense peaks indicative of FLBP modification are observed in the case of synthesis in DMF. Initially, FLBP obtained in ACN and DMF did not show any chemical differences (Fig. 3d).

### Electrochemical properties of modified FLBP electrodes

From the studies carried out, the modifications can be divided into those that protect against degradation during electrochemical processes with visible redox reactions in ferrocyanide, those that provide partial protection with a visible oxidation peak, and those that do not protect against FLBP oxidation. Some reactants cause blocking of the electrode surface or partially block the electrode surface, thus protecting or partially protecting against degradation, but do not allow electron exchange in the redox process occurring in the electrolyte, limiting the electrochemical applications of the electrode.

### Effect of isocyanate reactivity on protection against FLBP degradation

Isocyanates represent a highly reactive group of electrophilic species whose reactivity can be modulated by changing or modifying substituents outside the -NCO moiety. The order of reactivity of isocyanates used is approximately: alkyl-NCO < aryl-NCO < chloroalkyl-NCO < chlorocarbonyl-NCO < perchlorocarbonyl-NCO ≈ dichlorophosphoro-NCO. The introduction of electron-withdrawing groups, such as chlorine or carbonyl, enhances the affinity of -NCO for the nucleophilic phosphorus atoms in FLBP. As mentioned above, such an approach assumes the formation of an RNHC(O)-P < or RNHC(O)O-P < motif with the most active fragments on the FLBP surface. Thus, perchlorocarbonyl-NCO of dichlorophosphoro-NCO should form a thick layer on FLBP, whereas in the case of aryl or alkyl-NCO, insufficient coverage of active sites can be expected^56,57^. To explore an alternative binding strategy to the FLBP surface, isocyanates containing a chlorine moiety were also employed. The introduction of chlorine not only increases the reactivity of NCO but also allows the alkylation of FLBP via the Cl-alkyl moiety, leading to the formation of a phosphonium salt (≡ P^+^–R) on the surface of FLBP. This reaction pathway is particularly possible in the case of 4-(chloromethyl)phenyl isocyanate, moderately in the case of 2-chloroethylisocyanate or chloroacetyl isocyanate, and negligible in the case of trichloroacetyl isocyanate due to strong steric hindrance at the carbon atom^58,59^.

**Fig. 4:**
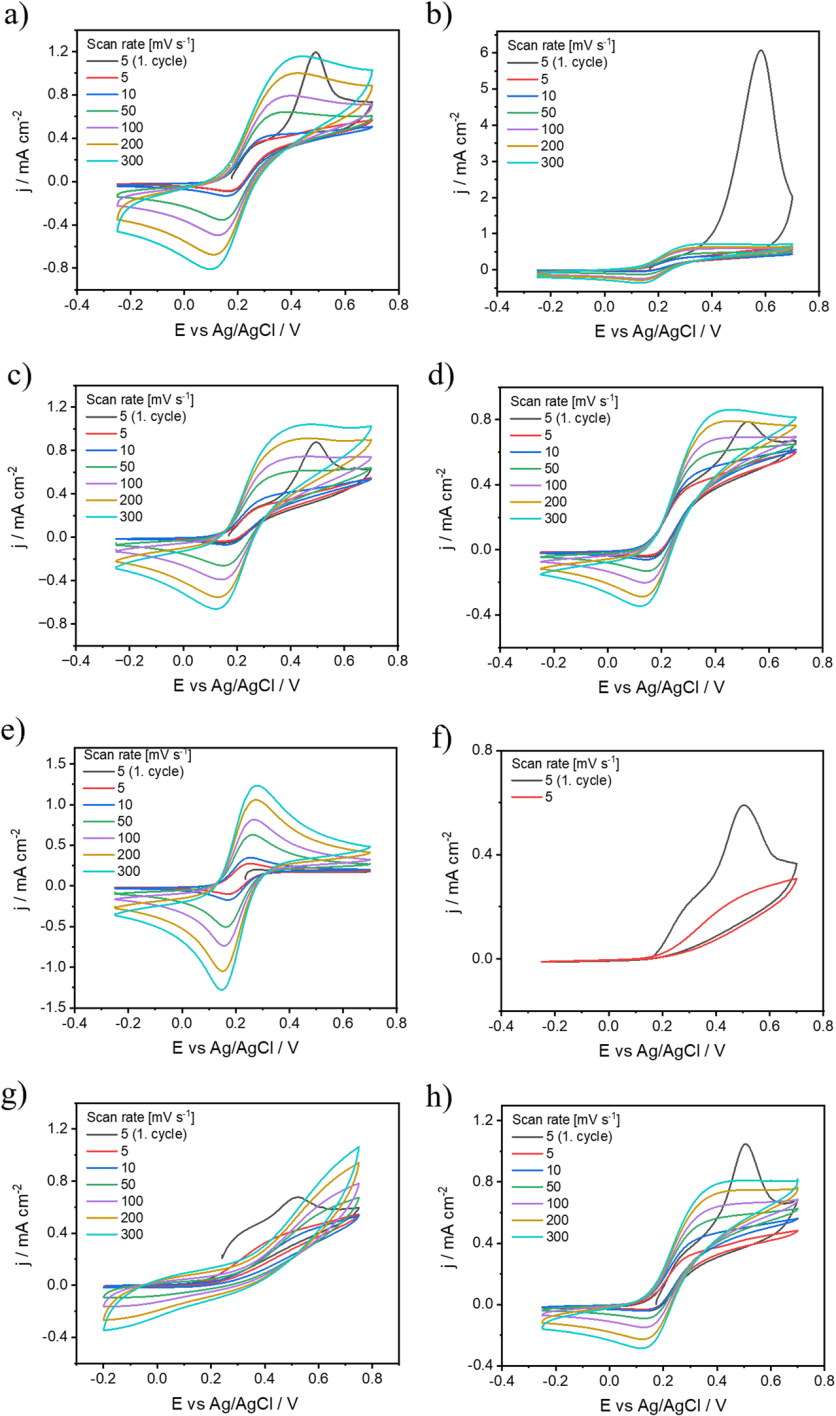
Cyclic voltammetry of FLBP modified with different chemical compounds (a) 1chB_DMF; (b) p-MphI_DMF; (c) EI_DMF; (d) 2chEI_mDMF; (e) 2xIP(O)(ch)_2__DMF; (f) chAI_DMF; (g) 2xchAch_DMF; (h) (ch)_3_AI_DMF ranked according by reactivity in 5 mM [Fe(CN)_6_]^3-/4-^ in 1 M KCl.

Due to the high affinity of RNCO for all nucleophilic impurities, a large excess of isocyanates had to be maintained during the process to ensure at least partial coverage of the FLBP surface. RNCO was used in quantities ranging from 0.5 to 2 equivalents relative to the total phosphorus (P) content of the sample. In this context, our investigation delves into the modification of FLBP using isocyanate, presents a novel approach to improving its stability and electrochemical properties and extends its potential utility in various applications such as electrochemical sensors, electronic devices. Figures 4a-h show cyclic voltammetry plots in 5 mM Fe(CN)_6_^3-/4-^ in 1 M KCl solution for FLBP electrodes modified with isocyanates, with the reactivity increasing from the lowest in Fig. 4a to the highest in Fig. 4h. However, the highest phosphorus oxidation peak is observed for FLBP modified with p-MphI_DMF. This suggests that the presence of the aromatic ring may hinder the reaction and provide ineffective protection against FLBP oxidation^60^. This is confirmed by the FTIR analysis of the modified FLBP (Fig. 3a), where no visible peaks are observed that would indicate the presence of functional groups from *p*-MphI_DMF. A visible, albeit reduced, oxidation peak is observed after modification with 1chB_DMF, EI_DMF, and 2chEI_mDMF, indicating that these FLBP modifications partially inhibit the oxidation process. However, the oxidation/reduction of Fe(CN)_6_^3-/4-^ remains unaffected, suggesting that the charge transfer process is not impaired^25^. The most effective modification appears to be the FLBP functionalised with 2xIP(O)(ch)_2__DMF, as the phosphorus oxidation peak is absent while the redox couple’s oxidation peak remains (see Fig. 4e). Effective protection against oxidation is consistent with the FTIR results, which show distinct peaks corresponding to the functional groups of 2xIP(O)(ch)_2__DMF (Fig. 3a). In contrast, subsequent modifications with chAI_DMF and 2xchAch_DMF (Fig. 4f and g), which involve more reactive compounds, reduced but did not eliminate the FLBP oxidation peak, indicating uneven coverage. However, the high reactivity caused a partial blocking of the electrode surface and a blocked charge transfer at the electrode/electrolyte interface, manifested by the absence of the Fe(CN)_6_^3-/4-^ oxidation peak^61^. The most reactive compound (ch)_3_AI_DMF did not block the surface and did not eliminate the oxidation of FLBP, but only reduced its oxidation^24^. In this case, referring to the FTIR analysis (Fig. 3a), no distinct peaks corresponding to (ch)_3_AI_DMF functional groups are observed after modification, further suggesting low functionalisation efficiency. The lower reactivity of (ch)_3_AI_DMF compared to expectations could be due to the way the reaction occurs at the surface of the material. The spherical barrier formed by the three chlorine atoms in the (ch)_3_AI_DMF molecule may have hindered its effective approach to the FLBP surface, limiting the reaction’s occurrence^60^.

#### Effect of FLBP-to-reactant ratio on protection against FLBP degradation

**Fig. 5:**
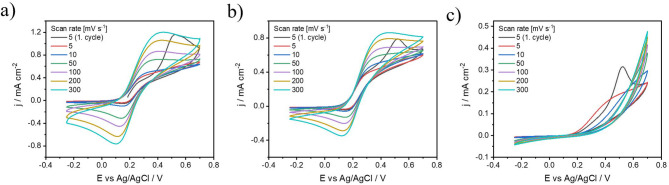
Effect of isocyanate concentration (2chEI_DMF) on protection against FLBP degradation, different FLBP: reactant ratios: (a) 2:1, (b) 1:1, (c) 1:2.

The extent of FLBP coverage is also affected by the reactant concentration^62,63^. Figure 5 illustrates the coverage of FLBP by 1chB_DMF at 3 different concentrations: the stoichiometric ratio of FLBP-to-reactant of 1:0.5, an equilibrium ratio of 1:1, and an excess reactant condition with 1:2 ratio. The electrochemical properties of modified FLBP films exhibit measurable changes with varying FLBP/reactant ratios. As the ratio shifts from 2:1 to 1:1, cyclic voltammetry shows a reduction in the oxidation current for the redox probe from 1.2 mA cm^−2^ (Fig. 5a) to 0.85 mA cm^−2^ (Fig. 5b) at the highest scan rate. At the same time, the peak current for phosphorus oxidation decreases from 1.15 mA cm^−2^ to 0.8 mA cm^−2^. Further reduction of the FLBP content to a 1:2 ratio suppresses the FLBP oxidation effect to approximately 0.3 mA cm^−2^. Notably, this composition also significantly impairs electron transfer during the ferrocyanide redox process, as evidenced by the absence of discernible redox peaks in the corresponding voltammogram (Fig. 5c)^24^.

### Influence of ultrasonic energy on FLBP electrode properties

**Fig. 6:**
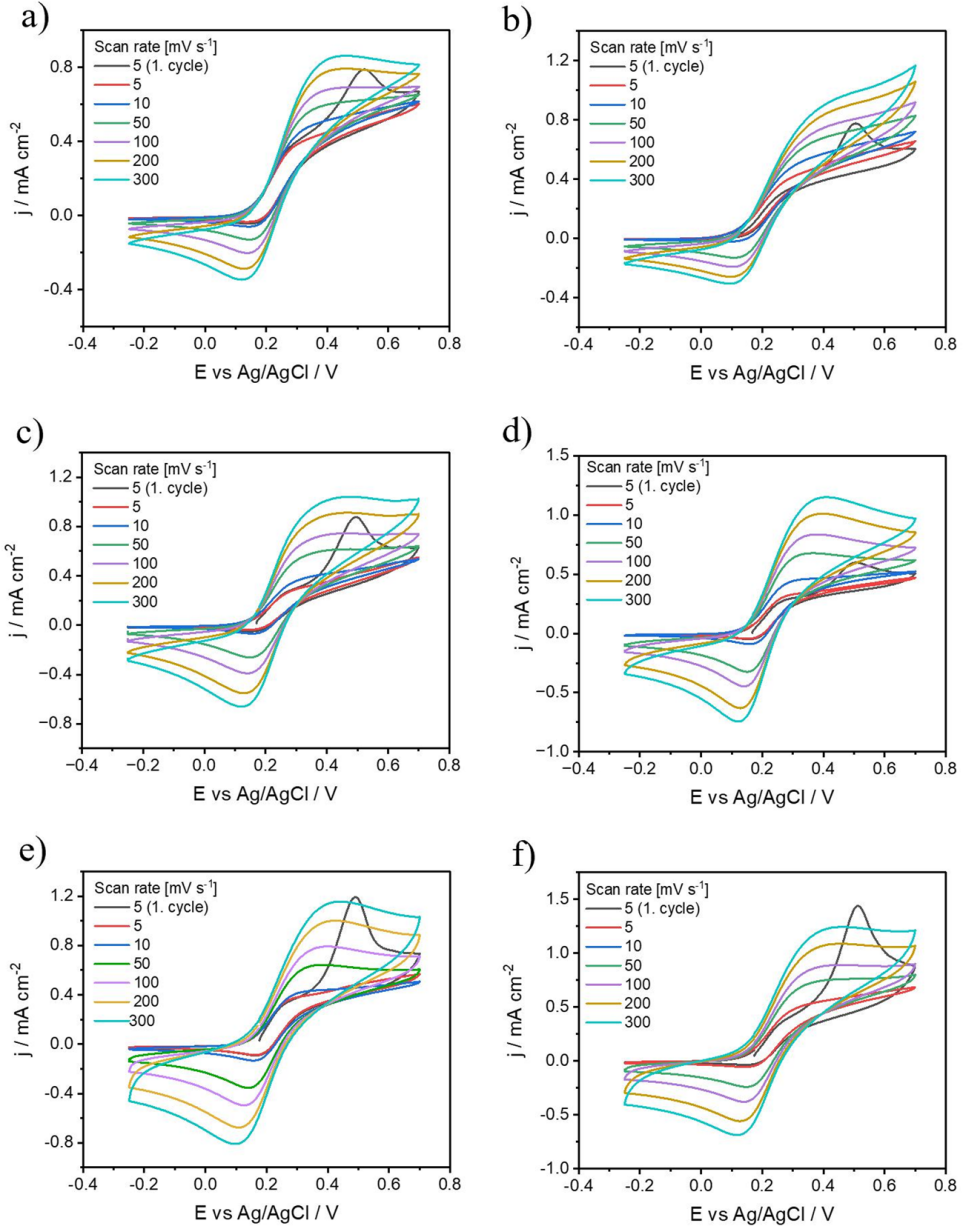
Cyclic voltammetry of modified FLBP with (a) 2chEI_mDMF; (c)EI_mDMF; (e)1chB_mDMF; and additionally with ultrasound (b) 2chEI_mDMF_US; (d) EI_mDMF_US; (f)1chB_mDMF_US in 5 mM [Fe(CN)_6_]^3−/4−^ in 1 M KCl.

The next step was to investigate the impact of ultrasonic energy on the properties of the modified FLBP electrode^64^. Figure 6 illustrates how ultrasonic energy influences three different reagents, 2-chloroethyl isocyanate, ethyl isocyanate, and 1-chlorobutane, which vary in reactivity. For the least reactive reagent, 1-chlorobutane, no significant differences were observed between modifications performed with or without ultrasound (Fig. 6e-f). The sizes of the FLBP oxidation peaks for 1chB_mDMF and 1chB_mDMF_US were comparable. FTIR analysis indicates that no reaction likely occurs in this case, and ultrasonication does not influence the process. Therefore, factors such as increased active sites or improved mass transport do not affect the modification process when the reagent is non-reactive^65^. In contrast, for the more reactive reagents (2-chloroethyl isocyanate and ethyl isocyanate) (Fig. 6a and c), where FTIR analysis confirmed the reaction, the application of ultrasonic energy during modification resulted in a smaller FLBP oxidation peak compared to the redox pair oxidation peak^25^. This suggests that ultrasonication improves protection against electrode material degradation (Fig. 6b and d). The enhancement can be attributed to more efficient reactions, enabled by improved mass transport and a higher density of active sites^66^. Additionally, for the 2chEI_mDMF_US and EI_mDMF_US modifications, the oxidation and reduction peaks of the redox pair slightly increased, which may be related to the greater number of active sites created by ultrasonication^67^. Specifically, for 2chEI_mDMF, the oxidation peak of the redox pair increased from 0.85 mA cm⁻² to 1.1 mA cm⁻², and for EI_mDMF, it increased from 1 mA cm⁻² to 1.2 mA cm⁻².

### Effect of solvent type on the properties of the FLBP protective layer

**Fig. 7:**
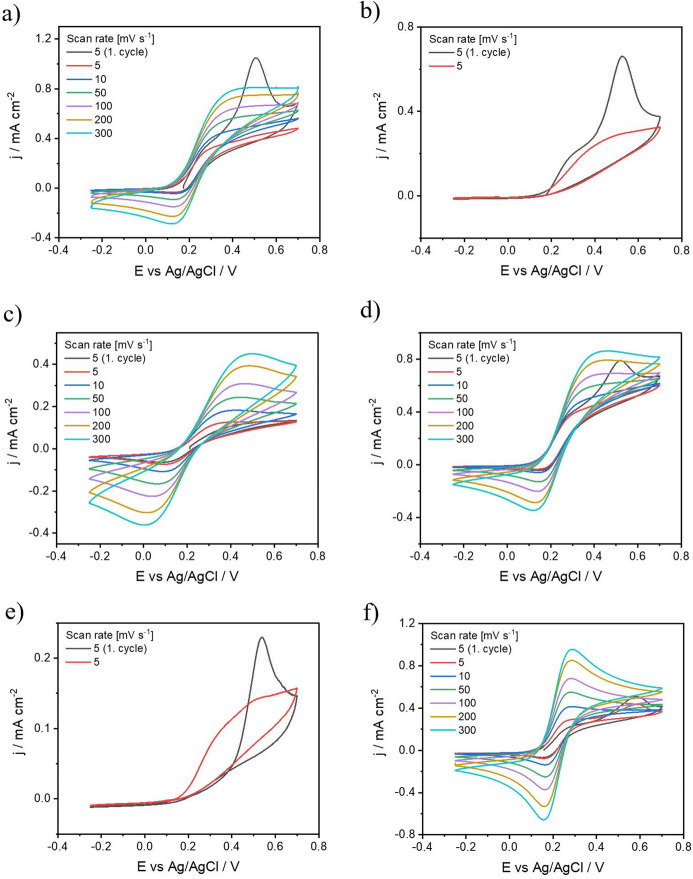
Cyclic voltammetry of modified FLBP with (a) (ch)_3_AI_DMF; (b) 2x(ch)_3_AI_DMF; (c) 2x(ch)_3_AI_ACN; (d) 2chEI_DMF; (e) 2 × 2chEI_DMF and (f) 2 × 2chEI_ACN in 5 mM [Fe(CN)_6_]^3−/4−^ in 1 M KCl.

Figure 7 shows the effect of solvent on the FLBP modification process. Two different solvents were used during the experiment: the more polar DMF and the less polar aprotic ACN. ACN, as a less polar aprotic solvent, may promote different reaction mechanisms^68^. Its structure favours interactions with isocyanates, but it may not be as effective as DMF in stabilising intermediates^69^. When reacting with isocyanates, ACN can provide effective protection against hydrolysis of the isocyanate groups, which is advantageous in the context of syntheses requiring longer reaction times without substrate degradation^70^. For reactions conducted in DMF, it was observed that the modification did not result in full encapsulation of the FLBP surface for either reactant. This is evidenced by the appearance of an oxidation peak within the 0.5–0.6 V potential range (Fig. 7a, d), even in the presence of an excess amount of reactant (Fig. 7b, e). In addition, a deterioration in the electrochemical properties and a decrease in the charge exchange efficiency of the redox processes for Fe(CN)_6_^3-/4-^ were observed with an excess of reactant in DMF. In contrast, the reactions carried out in ACN, using both reactants in excess, effectively protected FLBP from oxidation during the electrode process at potentials below 0.7 V (Fig. 7c, f). For the 2x(ch)_3_AI_ACN sample, a reduction in current density was observed for Fe(CN)_6_^3-/4-^ oxidation, while maintaining a comparable separation of oxidation and reduction peaks in the redox couple. In contrast, in the case of the 2 × 2chEI_ACN modification (Fig. 7f), a significant improvement in electrochemical properties was obtained compared to the corresponding synthesis in DMF (Fig. 7d) with an equivalent amount of reactant. An increase in redox pair oxidation current from 0.8 mA cm^−2^ to 1 mA cm^−2^ and a decrease in peak separation from 191 mV to 131 mV at a scan rate of 300 mV s^−1^ were observed. These results indicate a reduction in charge transfer resistance.

### Electrochemical properties of FLBP modified by isocyanatophosphonic dichloride

**Fig. 8:**
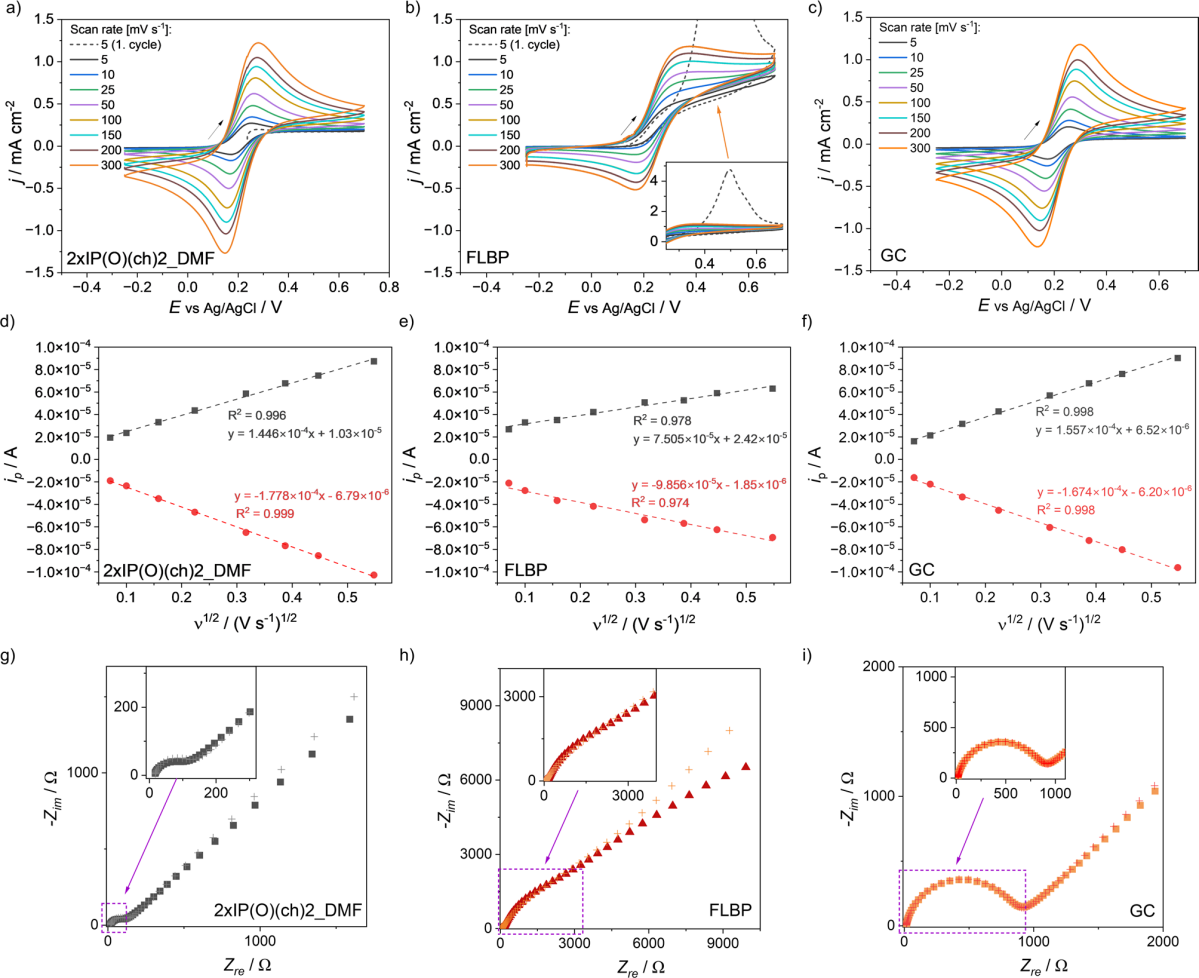
Electrochemical behaviour of electrodes recorded in 5 mM [Fe(CN)_6_]^3−/4−^ in 1 M KCl: cyclic voltammetry of (a) FLBP modified with 2xIP(O)(ch)_2_, (b) FLBP, (c) pure GC; dependence of the peak current on the scan rate (based on the results shown in Fig. 8a-c) of (d) FLBP modified with 2xIP(O)(ch)_2_, (e) FLBP, (f) pure GC; Nyquist plot representation of the impedance of (g) FLBP modified with 2xIP(O)(ch)_2_, (h) FLBP, (i) pure GC (cross symbols represent the fitted EIS data).

The modification with isocyanatophosphonic dichloride (2xIP(O)(ch)_2__DMF) not only protected against oxidation but also enhanced the electrochemical properties when compared to FLBP. The electrochemical behaviour of FLBP and modified FLBP was thoroughly investigated using CV and EIS techniques (see Fig. 8a-c and g-i). The study of electrochemical kinetics focused on the inner-sphere electron transfer process of the Fe(CN)_6_^3-/4-^ redox mediator^71^. The CV curves obtained from this redox system allow for the determination of electrode reaction kinetics (Fig. 8d-f). This allows the measurement of electron transfer reactions through both modified and unmodified FLBP electrodes. The estimated electrochemical parameters are summarised in Table 2. The 2xIP(O)(ch)_2__DMF electrode exhibits a lower peak-to-peak separation (Δ*E*_*p*_ = 86.7 ± 11.1 mV) compared to the FLBP electrode, which has value of 133.6 ± 4.6 mV. This suggests faster electron transfer kinetics for the modified 2xIP(O)(ch)_2__DMF electrode, associated with easier adsorption of electroactive ions on the surface of the 2xIP(O)(ch)_2__DMF electrode and more efficient electron transfer compared to FLBP electrode^45^. The oxidation and reduction currents are slightly higher for the 2xIP(O)(ch)_2__DMF electrode (*j*_*p, ox*_ = 0.76 mA cm^−2^; *j*_*p, red*_ = 0.73 mA cm^−2^) compared to FLBP (*j*_*p, ox*_ = 0.64 mA cm^−2^; *j*_*p, red*_ = 0.62 mA cm^−2^) electrode.

The next step was to estimate the value of the parameter proposed by Matsuda and Ayabe, Λ, together with the heterogeneous electron transfer (HET) rate constant, which helps to categorise the type of electrochemical reaction (the calculation method is detailed in SI 7)^72,73^. The 2xIP(O)(ch)_2__DMF electrode exhibits a higher apparent heterogeneous rate constant (*k*^*0*^) of 5.3 × 10^−3^ ± 0.7 × 10^−3^ cm s^−1^ compared to the unmodified FLBP (1.8 × 10^−3^ ± 0.2 × 10^−3^ cm s^−1^) electrode. This suggests that the modified 2xIP(O)(ch)_2__DMF layer facilitates better electron transfer thanunfunctionalised FLBP electrodes. The estimated Λ values are 1.37 ± 0.19 and 0.51 ± 0.04 for 2xIP(O)(ch)_2__DMF, and FLBP, respectively. For all electrodes, the Matsuda and Ayabe parameter falls within the quasi-reversible zone (15 > Λ > 10^−2(1+α)^), where, α represents the transfer coefficient, which typically ranges from 0 to 1 depending on the symmetry of the energy barrier for electron transfer^74^, indicating quasi-reversible redox reactions of the [Fe(CN)_6_]^3-/4-^ pair at a scan rate of 50 mV s^−1^. This suggests that both mass transport and charge transfer influence the electrode reaction^73^. However, the parameters for the functionalised electrode indicate faster electrode kinetics than for reactions FLBP electrodes. Other parameters also support the quasi-reversible nature of the reaction. The kinetic parameter ψ for 2xIP(O)(ch)_2__DMF is 0.77 ± 0.10, which is lower than 1 (ψ = 1 indicates reversible reactions) but higher than 0.001, the value for irreversible reactions. Therefore, ψ for 2xIP(O)(ch)_2__DMF is in the range of semi-reversible reactions^73^.

In addition, the previously calculated *k*^*0*^ for 2xIP(O)(ch)_2__DMF, GC and FLBP electrodes (5.3 × 10^−3^ cm s^−1^; 4.9 × 10^−3^ cm s and 1.8 × 10^−3^ cm s^−1^ respectively) are in the quasi-reversible range, between 0.02 cm s^-1^ for reversible reactions and 5 × 10^−5^ cm s^−1^ for irreversible reactions (*T* = 298 K, ν = 0.1 V s^−1^, *D* = 7.6 × 10^−6^ cm^2^ s^−1^, α = 0.5)^73,75,76^. Figures 8d-f show the relationship between the anodic and cathodic peak currents and the square root of the scan rate, as extracted from Fig. 8a-c. A linear correlation between *i*_*p*_ and ν^1/2^ is observed for 2xIP(O)(ch)_2__DMF, GC and FLBP electrodes, indicating diffusion-controlled charge transfer kinetics for both oxidation and reduction processes^77^. As observed, both the cathodic and anodic peak currents increased with the sweep rate. In addition, the oxidation and reduction peaks exhibit symmetry, indicating a nearly reversible electron transfer reaction^75^. The highest slope values were recorded for the GC electrode (1.557 × 10^−4^ A; −1.674 × 10^−4^ A), 2xIP(O)(ch)₂_DMF (1.446 × 10^−4^ A; −1.778 × 10^−4^ A), and FLBP (7.505 × 10^−5^ A; −9.856 × 10^−5^ A).

**Table 2:** Average electrochemical properties of investigated modified FLBP (2xIP(O)(ch)_2__DMF), FLBP and GC electrodes (for 50 mV s^−1^; *n* = 3).

	2xIP(O)(ch)2_DMF	FLBP	GC
*E*_*p, ox*_/mV	268.5 ± 9.8	306.3 ± 17.9	263.7 ± 2.9
*j*_*p, ox*_/mA cm^−2^	0.76 ± 0.10	0.64 ± 0.03	0.67 ± 0.05
*E*_*p, red*_/mV	181.7 ± 12.4	172.7 ± 13.9	169.8 ± 4.5
*j*_*p, red*_/mA cm^−2^	0.73 ± 0.05	0.62 ± 0.03	0.69 ± 0.04
*j*_*p, red*_/*j*_*p, ox*_	0.97 ± 0.08	0.97 ± 0.03	1.04 ± 0.02
*ΔE*_*p*_/mV	86.7 ± 11.1	133.6 ± 4.6	93.9 ± 6.3
*R*_*ct*_/Ω	98 ± 30	3059 ± 1214	522 ± 177
*Λ*	1.37 ± 0.19	0.51 ± 0.04	1.26 ± 0.21
*Ψ*	0.77 ± 0.10	0.29 ± 0.02	0.71 ± 0.12
*k*^*0*^/cm s^−1^	5.3 × 10^−3^ ± 0.7 × 10^−3^	1.8 × 10^−3^ ± 0.2 × 10^−3^	4.9 × 10^−3^ ± 0.8 × 10^−3^
*A*_*e, ox*_/cm^2^	0.041 ± 0.002	0.029 ± 0.006	0.047 ± 0.004
*A*_*e, red*_/cm^2^	0.054 ± 0.002	0.029 ± 0.002	0.051 ± 0.004

These results suggest that surface modification enhances charge transfer, leading to a higher slope for the 2xIP(O)(ch)_2__DMF electrode compared to the FLBP electrode. The electrochemical behaviour of the prepared 2xIP(O)(ch)_2__DMF, GC, and FLBP electrodes was further characterised using EIS. The impedance spectra were fitted to electrical equivalent circuit (EEQC) based on the model in Figure S2, SI 8. This model includes a constant phase element (CPE) in parallel with the charge transfer resistance (*R*_*ct*_) at the electrode/electrolyte interface. The Warburg impedance (*W*) was included in series with *R*_*ct*_ to represent the diffusion impedance. The components representing the GC, FLBP and 2xIP(O)(ch)_2__DMF electrodes were placed in series with a resistor (*R*_*e*_), to account for the resistivity of the electrolyte solution^72,78^. The results of the EEQC fitting are summarised in Table S2, SI 8. The Nyquist plots (Fig. 8g–i) display a semicircle in the mid to high-frequency range, corresponding to the *R*_*ct*_ of the electrodes^72^. The 2xIP(O)(ch)₂_DMF electrode exhibited a lower *R*_*ct*_, indicating reduced interfacial resistance for charge transfer between the electrode and the electrolyte. Modification of FLBP electrodes through the reaction of FLBP with 2xIP(O)(ch)_2_ in DMF enhanced the charge transfer kinetics and protected FLBP from oxidation. This is evident from the higher *R*_*ct*_ observed in unmodified FLBP compared to the GC and 2xIP(O)(ch)_2__DMF electrodes^24^. The linear region at lower frequencies in the impedance spectra corresponds to the semi-infinite linear diffusion impedance, known as *W*. Among the tested electrodes, the GCE exhibited the lowest Warburg impedance, as reflected by the shortest 45º linear part, suggesting superior ion diffusion kinetics due to its minimal surface area^72,78^. However, a significant improvement in *W* was observed for the FLBP electrode after modification with 2xIP(O)(ch)_2_ in DMF. The modified electrode showed a shorter linear part at low frequencies compared to unmodified FLBP, indicating enhanced stability and ion transport. In contrast, the FLBP electrode exhibited curvature in the low-frequency region, suggesting sample instability during testing, likely due to degradation caused by interactions of oxidised FLBP with the aqueous electrolyte^24^. As a control, EIS was also performed in supporting electrolyte (1 M KCl, without [Fe(CN)_6_]^3-/4-^). The spectra (Fig. S3, SI 8) showed only the solution resistance and double-layer capacitance, without a semicircle corresponding to charge-transfer resistance. This confirms that the semicircle observed in the presence of the ferri/ferrocyanide mediator arises from a faradaic electron-transfer process rather than artefacts associated with electrode porosity or contact resistance.

Electrochemical characterisation using CV and EIS consistently indicates that 2xIP(O)(ch)_2__DMF-modified FLBP exhibits superior charge transfer properties compared to pristine FLBP. In CV, the modified electrode shows a significantly reduced peak-to-peak separation (Δ*E*_*p*_ = 86.7 ± 11.1 mV vs. 133.6 ± 4.6 mV for unmodified FLBP) and a higher heterogeneous electron transfer rate constant (*k*^*0*^ = 5.3 × 10^−3^ ± 0.7 × 10^−3^ cm s^−1^ vs. 1.8 × 10^−3^ ± 0.2 × 10^−3^ cm s^−1^), values that are statistically comparable to those obtained for bare GC. In parallel, EIS measurements (at open circuit potential, OCP-EIS) yield an equilibrium *R*_*ct*_ of 98 ± 30 Ω for 2xIP(O)(ch)_2__DMF, which is substantially lower than for both pristine FLBP (3059 ± 1214 Ω) and GC (522 ± 177 Ω). This ranking (2xIP(O)(ch)_2__DMF < GC < FLBP) demonstrates that surface functionalisation with isocyanatophosphonic dichloride not only protects FLBP against oxidation but also facilitates faster interfacial electron transfer. We note that absolute values of *R*_*ct*_ from EIS and kinetic parameters from CV are not directly comparable because they probe different regimes: EIS at OCP captures the equilibrium exchange resistance, whereas CV reflects the differential kinetics under anodic polarization^79,80^. Nevertheless, both techniques converge on the same conclusion: the functionalised electrode achieves interfacial charge transfer kinetics at least on par with GC, and in terms of equilibrium resistance even surpasses it.

## Conclusions

The study highlights the impact of isocyanate-based modifications on the electrochemical properties and stability of FLBP electrodes. The results demonstrate that different isocyanate reactants vary in their ability to protect FLBP from oxidation, with the most effective modification being 2xIP(O)(ch)_2__DMF. This modification not only inhibits phosphorus oxidation but also preserves redox activity, thereby enhancing overall electrochemical performance. Key findings include the role of reactant concentration, ultrasonic energy, and solvent type in influencing the degree of FLBP protection. Higher reactant concentrations improve surface coverage but can impede charge transfer, whereas ultrasound treatment promotes better reactant interaction and enhances FLBP stability. Additionally, the choice of solvent plays a crucial role, with ACN proving to be more effective than DMF in maintaining electrochemical properties and ensuring better protection against FLBP degradation.

Electrochemical characterisation using CV and EIS consistently confirms that 2xIP(O)(ch)_2__DMF-modified FLBP exhibits superior charge transfer kinetics compared to pristine FLBP. CV shows a significantly reduced peak-to-peak separation and higher heterogeneous electron transfer rate constant, values statistically comparable to bare glassy carbon (GC). In parallel, OCP-EIS reveals the lowest equilibrium charge-transfer resistance for the modified electrode (98 Ω), substantially lower than both GC (522 Ω) and unmodified FLBP (3059 Ω). While CV and EIS probe different regimes of electrode kinetics, both converge on the same conclusion: functionalisation raises the charge-transfer performance of FLBP to the level of GC and, in terms of equilibrium resistance, even surpasses it.

Overall, this research provides valuable insights into the optimisation of FLBP modifications using isocyanates, offering a promising approach for enhancing the stability and functionality of FLBP-based electrochemical systems. Future studies may further explore the practical applications of these modified electrodes in energy storage, catalysis, and sensor technologies.

## Supplementary Information

Below is the link to the electronic supplementary material.

Supplementary Material 1

## Data Availability

Measurement data supporting this study’s results have been deposited in the Bridge of Knowledge (https://mostwiedzy.pl/en/?) and assigned the number doi: 10.34808/98a9-vq18.
