# Supplementary material for: Effect of covalent functionalisation with isocyanates on the electrochemical properties of exfoliated black phosphorus electrodes
Source: Sci Rep. 2025 Oct 16;15:36261. doi: 10.1038/s41598-025-20117-3 (PMC12533256; doi:10.1038/s41598-025-20117-3)
Supplement: Supplementary file 1 — Supplementary Material 1 [file 41598_2025_20117_MOESM1_ESM.docx]

**Supplementary Information**

Effect of covalent functionalisation with isocyanates on the electrochemical properties of exfoliated black phosphorus electrodes

Paweł Jakóbczyk,^1*^ Anna Dettlaff^1,2^, Mattia Pierpaoli^1^, Barbara Wójcik^3^, Sławomir Makowiec^2^, Robert Bogdanowicz^1^

*^1^Gdańsk University of Technology, Faculty of Electronics, Telecommunications and Informatics, 11/12 Narutowicza Str., 80-233 Gdańsk, Poland*

*^2^Gdańsk University of Technology, Faculty of Chemistry, 11/12 Narutowicza Str., 80-233 Gdańsk, Poland*

*^3^Department of Nanobiotechnology, Warsaw University of Life Science, Ciszewskiego 8, 02-786 Warsaw, Poland*

*^*^Corresponding author: pawel.jakobczyk@pg.edu.pl*

**Table of Contents**

**SI 1:** Strategies of covalent modification of black phosphorus

**SI 2:** Spectral data of prepared azide and isocyanate

**SI 3:** Methods of modification

**SI 4:** Structures of reagents applied in FLBP surface functionalisation

**SI 5:** Abbreviations and naming scheme for modified FLBP electrodes

**SI 6:** Raman spectrum of FLBP and 2xIP(O)(ch)₂

**SI 7:** Calculation of electrochemical parameters

**SI 8:** EIS measurements

References

**SI 1: Strategies of covalent modification of black phosphorus**

**Table S1.** Comparative overview of covalent passivation strategies for black phosphorus

| Method | Reactive group | Target Site | P-P Bond Cleavage | Reaction Conditions | Bond Type | Stability | Functionals Tunability | Ref. |
| --- | --- | --- | --- | --- | --- | --- | --- | --- |
| Isocyanates | -N=C=O | Planar defects, edges | No | Mild, catalyst-free | P–N–C=O | High (chemical, thermal) | High | This work |
| Alkyl Halides (e.g. MeI) | R–X | Basal plane, edges | Yes | Sodium intercalation compounds | P–C or P–R | Moderate (prone to hydrolysis) | Moderate | 6 |
| Diazonium Salts | Ar–N₂⁺ | Edges, defect sites, basal plane | Partial | Catalyst free, diazonium salts can be thermally unstable | P–C (aryl) | High chemical stability | Limited | 7 |
| Azides | –N₃ | Basal plane | Yes | Elevated T | P=N | Moderate | \| Moderate \| \| --- \|  \|  \| \| --- \| | 8 |
| Radical-based addition | R• | Basal plane, edges, defect sites | No | Thermal initiation, Difficult to control | P–C | Low (structural damage) | High | 9 |
| Lewis acids (e.g. TiCL4) | Metal coordination | Lone pairs on surface P | No | \|  \| \| --- \|  \| Ambient, moisture sensitive \| \| --- \| | P→M (dative) | Moderate (reversible) | High | 10 |

"P–P Bond Cleavage": refers to undesired breaking of phosphorus lattice

"Functional Tunability": extent to which substituents or chemistry can be adapted

**SI 2: Spectral data of prepared azide and isocyanate**

1. 4-(chloromethyl)benzoyl azide


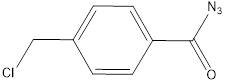


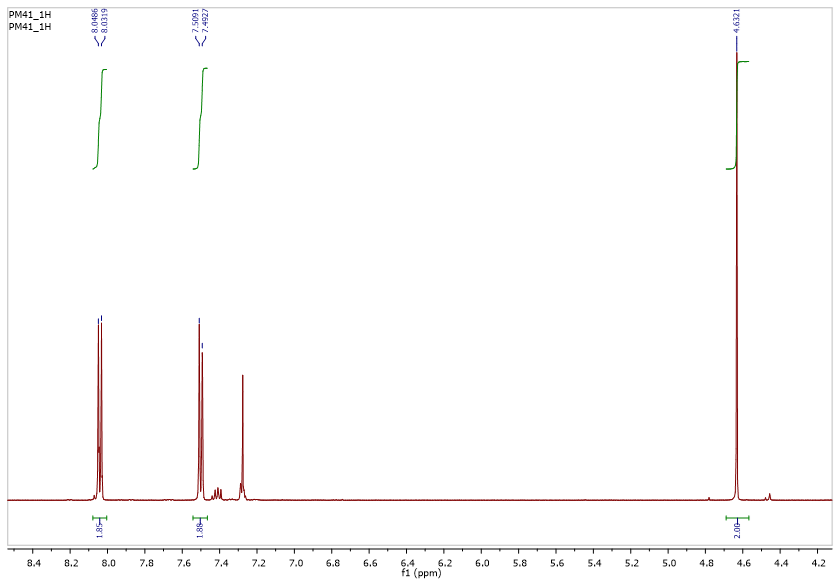


^1^H NMR spectrum of 4-(chloromethyl)benzoyl azide

^1^H NMR (CDCl_3_, 500 MHz): δ= 8.03 (d, J = 8.4 Hz, 2 H), 7.49 (d, J = 8.4 Hz, 2 H), 4.62 (s, 2 H)


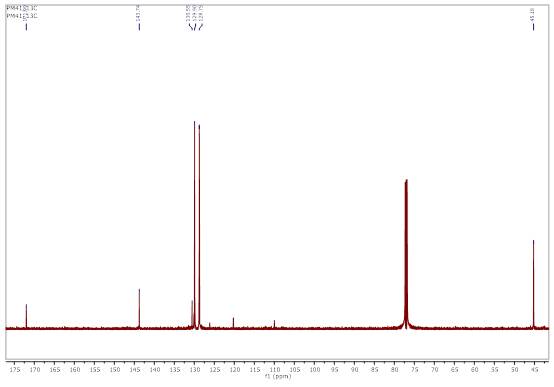


^13^C NMR spectrum of 4-(chloromethyl)benzoyl azide

^13^C NMR (CDCl_3_, 125 MHz): δ= 171.99, 143.74, 130.55, 129.90, 128.75, 45.18


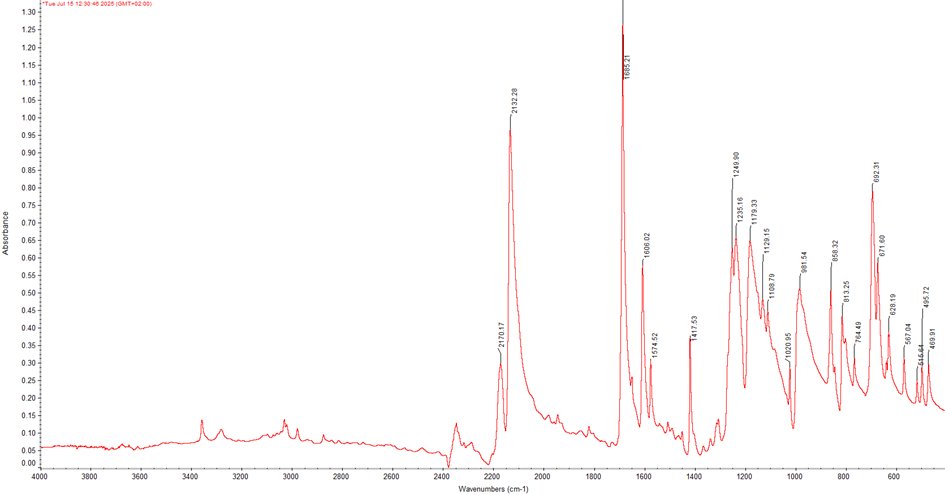


IR spectrum of 4-(chloromethyl)benzoyl azide

FT-IR (ATR) 2132 cm^-1^ N=N=N, 1685 cm^-1^ C=O

B). 4-(chloromethyl)phenyl isocyanate


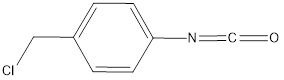


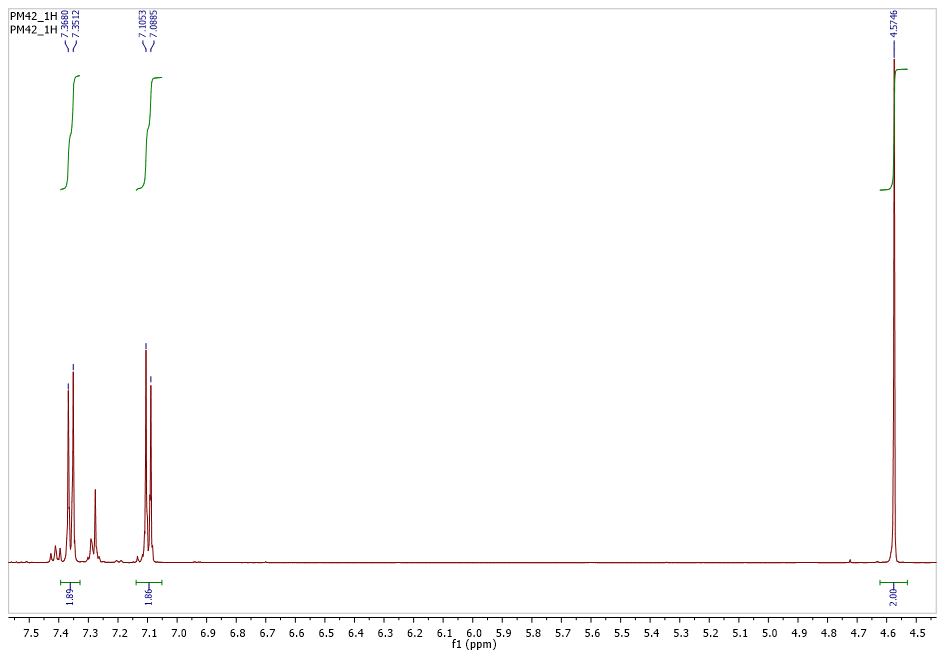


^1^H NMR spectrum of 4-(chloromethyl)phenyl isocyanate

^1^H NMR (CDCl_3_, 500 MHz): δ= 7.36 (d, J = 8.4 Hz, 2 H), 7.10 (d, J = 8.4 Hz, 2 H), 4.56 (s, 2 H)


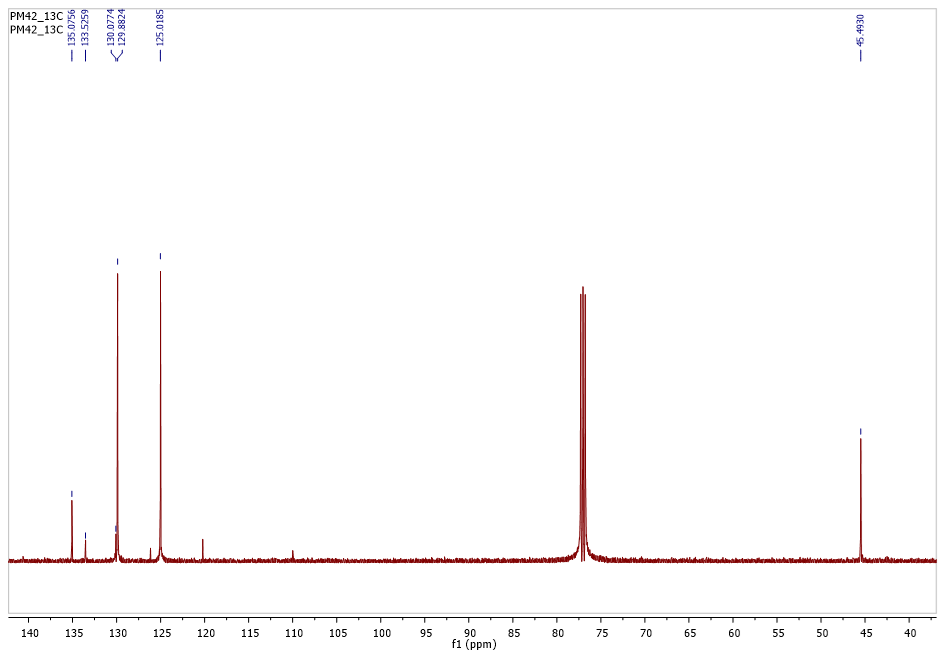


^13^C NMR spectrum of 4-(chloromethyl)phenyl isocyanate

^13^C NMR (CDCl_3_, 125 MHz): δ= 135.08, 133.53, 130.08, 129.87, 125.02, 45.49


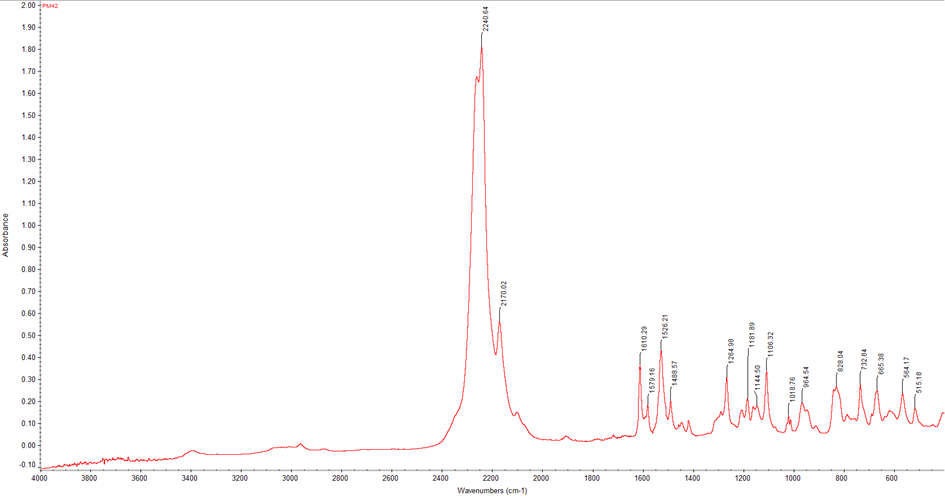


IR spectrum of 4-(chloromethyl)phenyl isocyanate

FT-IR (ATR) 2240 cm^-1^ N=C=O,

**SI 3: Methods of modification**

***Method A***

To the suspension of FLBP (9.88 mg, 0.32 mmol) in DMF (2 ml) under Argon, appropriate isocyanate (0.32 mmol) depicted in Table 1, was added and the resulting suspension was stirred for 24 h at R. T.

***Method A’***

To the suspension of FLBP (15.00 mg, 0.44 mmol) in DMF (4.7 ml) under Argon, appropriate isocyanate (0.44 mmol) depicted in Table 1 was added, and the resulting suspension was stirred for 24 h at R. T.

***Method B***

To the suspension of FLBP (8.4 mg, 0.27 mmol) in DMF (1.75 ml) under Argon, appropriate isocyanate (0.54 mmol) depicted in Table 1, was added. The resulting suspension was ultrasonicated for 3.5 h (120 W, 35 kHz) and stirred for 24 h at room temperature.

***Method C***

To the suspension of FLBP (8.4 mg, 0.27 mmol) in DMF (1.75 ml) under Argon, appropriate isocyanate (0.54 mmol) depicted in Table 1 was added, and the resulting suspension was stirred for 24 h at room temperature.

***Method D***

To the suspension of FLBP (15.00 mg, 0.44 mmol) in DMF (4.7 ml) under Argon, appropriate isocyanate (0.44 mmol) depicted in Table 1, was added. The resulting suspension was ultrasonicated for 3.5 h (120 W, 35 kHz) and stirred for 24 h at room temperature.

***Method E***

To the suspension of FLBP (5.5 mg, 0.18 mmol) in CH_3_CN (1.25 ml) under Argon, appropriate isocyanate (0.36 mmol) depicted in Table 1, was added and the resulting suspension was stirred for 24 h at room temperature.

***Method F***

To the suspension of FLBP (8.1 mg, 0.26 mmol) in DMF (1.5 ml) under Argon, appropriate isocyanate (0.13 mmol) depicted in the Table 1, was added and the resulting suspension was stirred for 24 h at room temperature.

**SI 4: Structures of reagents applied in FLBP surface functionalisation**


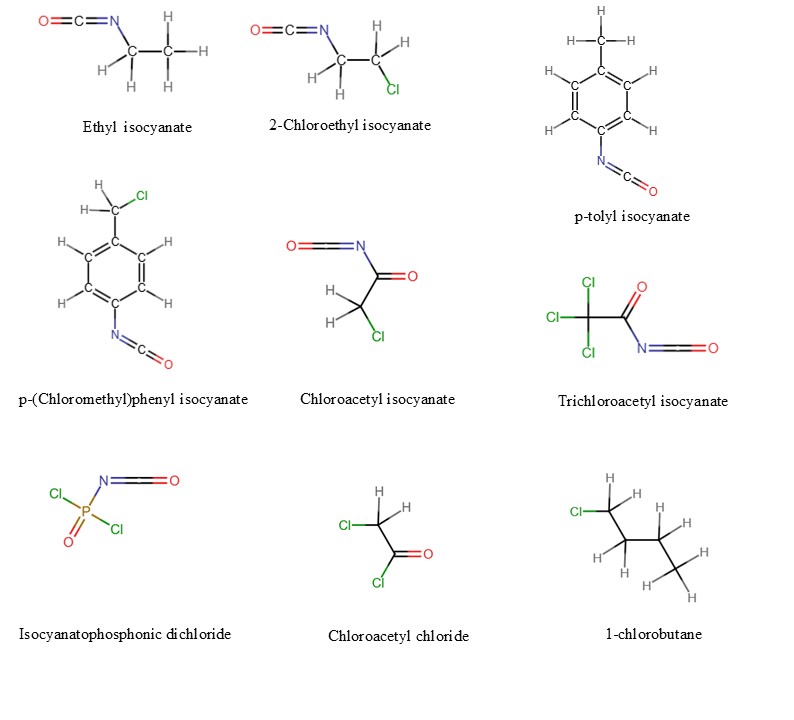


### **SI 5: Abbreviations and naming scheme for modified FLBP electrodes**

The abbreviations used for modified FLBP electrodes are systematically constructed to reflect both the chemical nature of the isocyanate modifier and the conditions of the modification procedure. Each name consists of a short identifier for the functionalising agent, followed by information on the solvent, stoichiometry, and whether ultrasonic treatment (US) was applied.

1. **Main component** (e.g., EI, 2chEI, p-MphI) refers to the type of isocyanate used (ethyl isocyanate, 2-chloroethyl isocyanate, *p*-tolyl isocyanate, respectively).
2. The **prefix** 2x or 0.5x denotes the molar ratio of isocyanate to FLBP (e.g., 2x = 2:1, 0.5x = 0.5:1).
3. The **solvent** is indicated by a suffix (e.g., _DMF, _ACN, _mDMF).
4. The addition of _US specifies that ultrasonic treatment was applied during modification.

**SI 6: Raman spectrum of FLBP and 2xIP(O)(ch)₂**


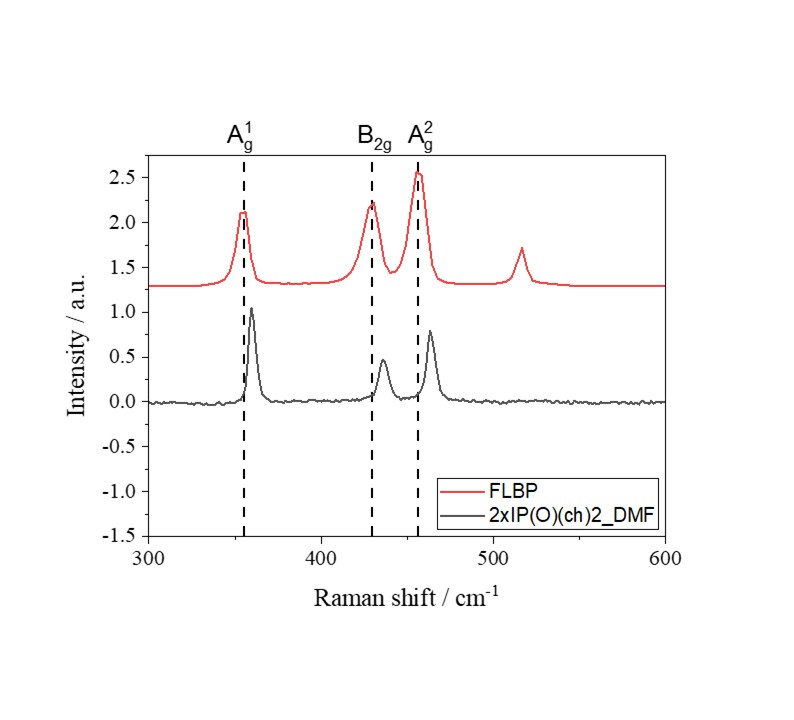


**Figure S1**. Raman spectrum of FLBP and 2xIP(O)(ch)₂

**SI 7: Calculation of electrochemical parameters**

The heterogeneous electron transfer (HET) rate constant *kº* [cm s^-1^] was calculated using the Eq. (1)^1^.

$k^{\circ}=\psi\cdot\sqrt{\frac{\pi\cdot D\cdot\nu\cdot F\cdot n}{R\cdot T}}$, (1)

where ψ is a kinetic parameter estimated based on the Δ*E* value by the Nicholson method (see Table SI 1.1)^2^, *D* signifies the diffusion coefficient (7.6·10^-6^ cm^2^ s^-1^)^3^, , ν [V s^-1^] is the scan rate, *F* indicates the Faraday constant (96,485 C), *n* is the number of electrons (1), *R* corresponds to the molar gas constant (8.314 J mol^-1^ K^-1^), *T* is the temperature (298 K).

**Table S1.** Kinetic parameter dependent upon the peak-to-peak separation value for a one-electron redox reaction (assuming α = 0.5)^2^.

| $\boldsymbol{\psi}$ | *ΔE_p_* / mV |
| --- | --- |
| 20 | 61 |
| 7 | 63 |
| 6 | 64 |
| 5 | 65 |
| 4 | 66 |
| 3 | 68 |
| 2 | 72 |
| 1 | 84 |
| 0.75 | 92 |
| 0.5 | 105 |
| 0.35 | 121 |
| 0.25 | 141 |
| 0.1 | 212 |

The parameter Λ was determined using Equation (2) (electron and ion) transfer at a liquid/liquid (L/L) interface, or at an oil/water (O/W) interface, or at an interface between two immiscible electrolyte solutions (ITIES) is one of the most fundamental physicochemical processes. When two electrically conducting phases such as w (water) and o (organic) are in contact, the partition of the charge carriers (electron and ion) between the two adjoining phases occurs due to the difference in energy of the carriers in both phases. Thus, an interfacial region is built up. The distribution of the potential at the interfacial region is related to the structure of the interface. One of the characteristics of electrochemistry at liquid/liquid interfaces is the diversity of charge transfer reactions which can be studied by electrochemical methodologies. These charge transfer reactions can be classified into three main categories: (a) ion transfer (IT) reaction; (b) facilitated ion transfer (FIT) reaction; (c) electron transfer (ET) reaction. Various methodologies and techniques such as the four-electrode system, two-electrode system, and three-electrode system may be used for electrochemical measurements at a liquid/liquid interface. Charge transfer reactions at liquid/ liquid interfaces find their application in pharmacokinetics, amperometric sensors, and electrochemical imaging. An important aspect of charge transfer reactions at a L/L interface is that all three types of transfer reactions can be employed to obtain imaging of various kinds of substrates and information about reactivity^1,3,4^:

$\Lambda=k^{\circ}\cdot\sqrt{\frac{R\cdot T}{F\cdot D\cdot\nu\cdot n}}$ , (2)

The electroactive surface area estimated for oxidation (*A_e,ox_*) and reduction reactions (*A_e,red_*) where calculated based on The Radles-Ševćik for quasi-reversible system (Eq. (3)).

$$i_{p}=0.4463\cdot\kappa\left( \Lambda,\alpha\right)\cdot n\cdot F\cdot A\cdot C\cdot D^{\frac{1}{2}}\cdot\nu^{\frac{1}{2}} \sqrt{\frac{n\cdot F}{\left( R\cdot T \right)}} (3)$$

where *κ(Λ,α*) is a introduced by Matsuda and Ayabe^5^ and it is estimated based on the value of logΛ with an assumption of α = 0.5.

**SI 8: EIS measurements**

Parameters obtained using electrochemical impedance spectroscopy was evaluated using equivalent circuit element and ZsimpWin software.


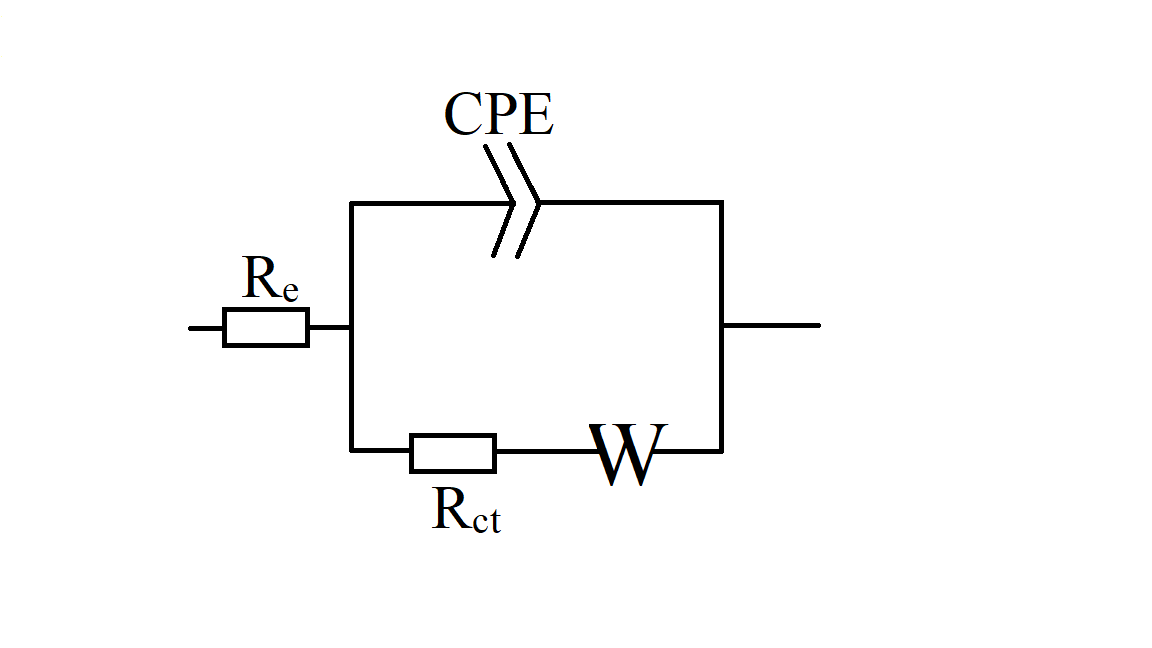


**Figure S2.** Electrical equivalent circuit (EEQC) used for EIS fitting.

**Table S2.** Fitted electrochemical impedance spectroscopy parameters obtained using the R(Q(RW)) equivalent circuit.

| Sample: | 2xIP(O)(ch)₂ | | FLBP | | GC | |
| --- | --- | --- | --- | --- | --- | --- |
|  | Calculated parameter | Relative standard error / % | Calculated parameter | Relative standard error / % | Calculated parameter | Relative standard error / % |
| *R*_e_/ Ω | 16.70 | 1.84 | 64.75 | 3.72 | 14.67 | 0.73 |
| *Q_2_* / Ω^-1^ s^n^ | 1.94 × 10^-6^ | 12.40 | 3.28 × 10^-5^ | 3.82 | 9.40 × 10^-7^ | 1.67 |
| *n_2_* | 0.89 | 1.33 | 0.68 | 2.26 | 0.89 | 0.18 |
| *R_ct1_* / Ω | 106 | 1.97 | 4763 | 12.79 | 842 | 0.31 |
| *s_2_ / Ω s^-0.5^* | 5.9 × 10^-4^ | 0.89 | 9.8 × 10^-5^ | 12.6 | 7.4 × 10^-4^ | 0.59 |
| ChiSqr χ^2^ | 7.2 × 10^-4^ |  | 1.9 × 10^-2^ |  | 1.4 × 10^-4^ |  |


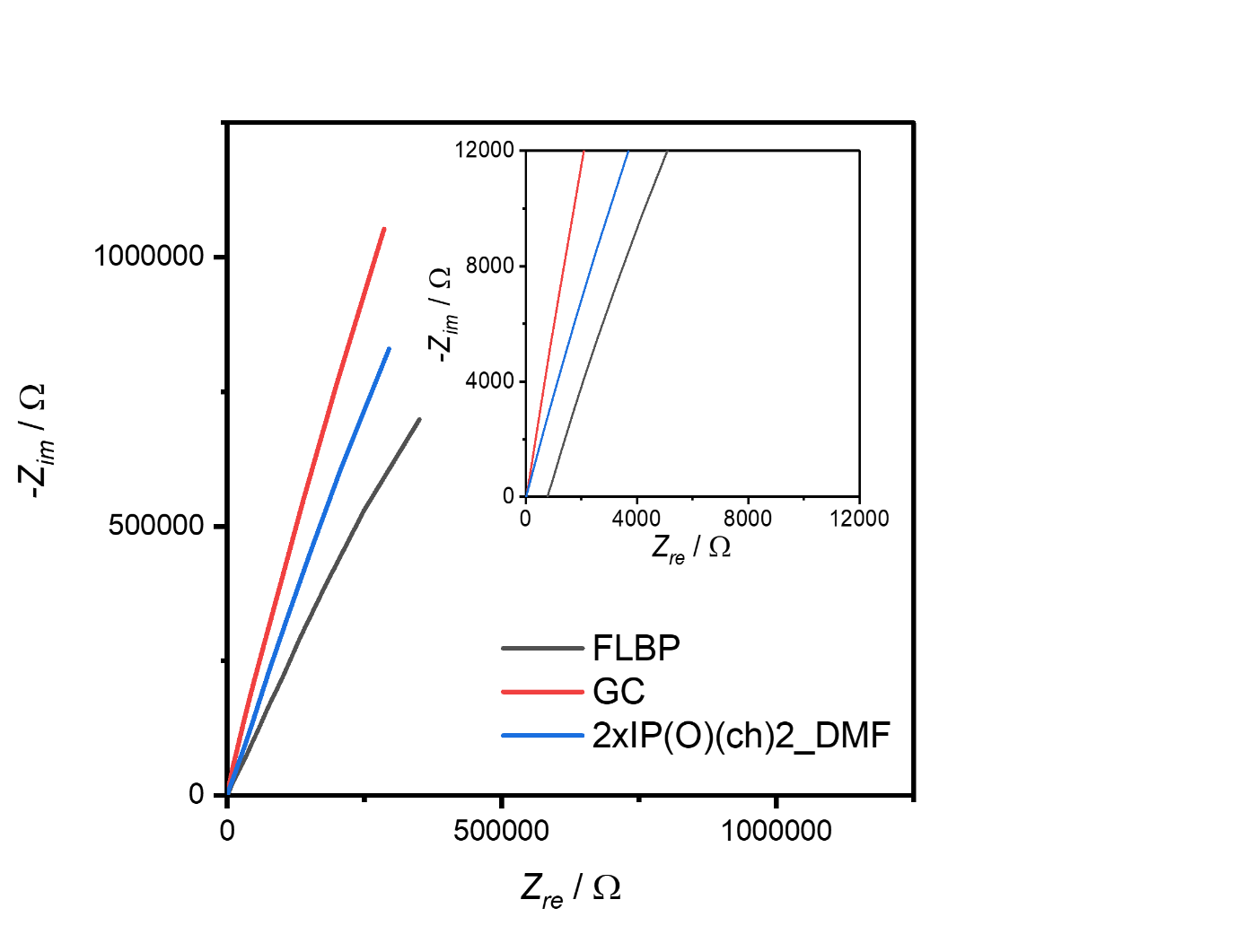


**Figure S3.** Nyquist plots of FLBP, 2xIP(O)(ch)₂_DMF, and GC electrodes recorded in supporting electrolyte (1 M KCl)

**References**

1. Zoski, C. G. *Handbook of Electrochemistry*. (Elsevier, 2007).

2. Nicholson, R. S. Theory and application of cyclic voltammetry for measurement of electrode reaction kinetics. *Anal. Chem.* **37**, 1351–1355 (1965).

3. Bard, A. J. & Faulkner, L. R. Electrochemical methods. fundamentals and appliactions. (John Wiley @ Sons, INC., 2001). doi:10.1016/j.aca.2010.06.020.

4. Compton, R. G. & Banks, C. E. Understanding voltammetry. (World Scientific, 2018).

5. Matsuda, H. & Ayabe, Y. Zur Theorie der Randles‐Sevčikschen Kathodenstrahl‐polarographie. Z. Für Elektrochem. Berichte Bunsenges*. Für Phys. Chem.* **59**, 494–503 (1955).

6. Wild, S. *et al.* Quantifying the covalent functionalization of black phosphorus. *Angew. Chem. Int. Ed.* **59**, 20230–20234 (2020).

7. Ryder, C. R. *et al.* Covalent functionalization and passivation of exfoliated black phosphorus via aryl diazonium chemistry. *Nat. Chem.* **8**, 597–602 (2016).

8. Liu, Y. *et al.* Azide passivation of black phosphorus nanosheets: Covalent functionalization affords ambient stability enhancement. *Angew. Chem. Int. Ed.* **58**, 1479–1483 (2019).

9. Hu, H. *et al.* Covalent functionalization of black phosphorus nanoflakes by carbon free radicals for durable air and water stability. *Nanoscale* **10**, 5834–5839 (2018).

10. Tofan, D. *et al.* Surface modification of black phosphorus with group 13 Lewis acids for ambient protection and electronic tuning. *Angew. Chem.* **133**, 8410–8417 (2021).
